# Fascia iliaca compartment block for surgical analgesia in children with osteogenesis imperfecta: A retrospective cohort study

**DOI:** 10.1371/journal.pone.0353186

**Published:** 2026-07-02

**Authors:** Yingxiang Xie, Long Zhang, Qing Yuan, Xueli Jiang, Qian Wang

**Affiliations:** 1 Department of Anesthesiology, Soochow University Affiliated Children’s Hospital, Suzhou, Jiangsu, China; 2 Department of Anesthesiology, Ningbo No. 6 Hospital, Ningbo, Zhejiang, China; Stanford University School of Medicine, UNITED STATES OF AMERICA

## Abstract

**Background:**

Osteogenesis imperfecta (OI) is a rare genetic bone disorder. Children with OI have a higher risk of intraoperative pain. The fascia iliaca compartment block (FICB) can reduce pain, improve intraoperative hemodynamic stability, and facilitate postoperative recovery. This study explores the feasibility and potential role of FICB in multimodal analgesia by describing its application experience in children with OI.

**Method:**

This study involved children with OI who underwent surgery for femoral shaft fractures. The FICB group received ultrasound-guided FICB combined with general anesthesia, whereas the control group received general anesthesia alone. Hemodynamic parameters, serum inflammatory markers, intraoperative opioid consumption, recovery time, postoperative pain scores, and the incidence of postoperative nausea and vomiting (PONV) were recorded.

**Results:**

A total of 126 children were enrolled. A significant time × group interaction was observed for changes in MAP and HR during surgery (*F*_3,372_ = 21.86 and *F*_3,372_ = 16.34, both *P* < 0.001). At all intraoperative time points, MAP and HR were lower in the FICB group than in the Control group (all *P* < 0.001). A significant time × group interaction was found for postoperative VAS scores (*F*_2,248_ = 3.18, *P* = 0.04); and they were lower in the FICB group at 2, 4, and 12 hours postoperatively (all *P* < 0.001). CRP levels also showed a significant time × group interaction (*F*_1,124_ = 290.3, *P* < 0.001); both groups exhibited elevated postoperative CRP versus baseline (*P* < 0.001), but the FICB group had lower levels. The FICB group had a shorter recovery time (*P* < 0.001), lower intraoperative fentanyl and remifentanil use (both *P* < 0.001), and a lower incidence of PONV (*P* = 0.026) compared to the Control group.

**Conclusion:**

In pediatric patients with OI undergoing femoral shaft fracture surgery, ultrasound-guided FICB has the potential to enhance intraoperative hemodynamic stability, alleviate postoperative pain, and decrease the incidence of adverse reactions, thereby facilitating the implementation of optimized multimodal analgesia.

## Introduction

Osteogenesis imperfecta (OI), an autosomal dominant genetic disorder, is characterized by defective collagen synthesis leading to abnormal bone development [1]. Clinically, it manifests as increased bone fragility, recurrent fractures, short stature, limb deformities, joint ligament laxity, and hearing loss, among other multi-system involvements. Femoral shaft fractures are common in patients with OI. Surgical reduction of such fractures entails prolonged operative time and substantial tissue trauma, which can significantly affect respiratory and circulatory function, thereby posing multiple challenges for intraoperative anesthesia management [2].

In the surgical treatment of femoral shaft fractures in patients with OI, pain management poses particular challenges due to their fragility, recurrent fractures, potential chronic pain, and high anesthesia risks [2]. Traditional anesthesia methods often cause significant hemodynamic fluctuations due to insufficient analgesia, thereby increasing intraoperative risks [3]. The combined application of general anesthesia and regional nerve blockade has demonstrated unique value in various surgeries, especially for complex surgeries or patients with underlying diseases. This approach achieves this through stress inhibition, optimization of drug dosage, organ protection, and reduction of postoperative adverse reactions, thus becoming an effective anesthesia method. Therefore, it is of great clinical significance to explore a regional anesthesia technique that can reduce the dosage of opioids, respiratory depression, and fracture risk.

The fascia iliaca compartment block (FICB), as a regional anesthesia approach, can effectively encompass the surgical region of the femoral shaft through the blockade of nerve conduction in the branches of the lumbar plexus. This leads to a reduction in the dosage of general anesthetic agents, consequently mitigating the risks of respiratory and circulatory depression [4]. Recent studies have shown that ultrasound-guided FICB can accurately locate the nerve trajectory and improve the success rate of blockage, especially suitable for special populations such as children [5].

Currently, research on anesthesia interventions for children with OI, a special population, remains limited, particularly high-quality descriptive reports on surgical procedures involving long bones of the extremities [6,7]. Therefore, a systematic evaluation of the actual efficacy of different anesthesia strategies in such patients not only helps fill existing evidence gaps but also provides essential support for establishing individualized, safe, and effective anesthesia practice guidelines. This study aims to compare the efficacy of ultrasound-guided femoral nerve block combined with a laryngeal mask airway (LMA) general anesthesia versus LMA general anesthesia alone in pediatric patients with OI undergoing femoral shaft fracture surgery. By describing the application experience of FICB in children with OI, the feasibility and potential role of FICB in multimodal analgesia are explored, providing clinical evidence for optimizing perioperative anesthesia management strategies in this patient population.

## Materials and methods

### Involvement

This study retrospectively analyzed data from patients who underwent surgical treatment for femoral shaft fractures caused by OI at the Children’s Hospital Affiliated to Soochow University between 01/01/2018 and 31/12/2023. The study received approval from the Ethics Committee of the Children’s Hospital Affiliated to Soochow University (Approval No. 2024CS178), and informed consent was waived. Data for this study were accessed for research purposes on 30/11/2024.

Inclusion criteria encompassed children aged between 6 and 15 years, with a classification of I or II according to the American Society of Anesthesiologists (ASA), no preoperative respiratory or cardiovascular diseases, normal hepatic and renal functions, and no neuromuscular disorders. Exclusion criteria included children whose medical records were seriously incomplete, those with a history of local anesthetic allergy, cognitive impairment, coagulation dysfunction, contraindications to surgery or anesthesia, and those with cardiovascular disease or multiple organ dysfunction.

Patients were classified into two groups according to anesthesia modality: the FICB group and the control group. The FICB group received ultrasound-guided FICB in combination with LMA general anesthesia, while the Control group underwent LMA general anesthesia alone. Prior to surgery, anesthesiologists determine whether a patient is suitable for FICB based on comprehensive factors such as the patient’s specific condition, and thoroughly communicate with the patient and their parents. The final choice of analgesic method is entirely determined by the patient and their parents. All clinical data for the patients in this study were retrospectively collected from medical records, and the anesthesiologists’ decision-making process was based on clinical experience and the patient’s actual condition, without any intervention by the investigators. All patients complied with preoperative fasting and fluid restriction protocols. Upon entering the operating room, patients were given face-mask oxygen inhalation, and continuous monitoring of electrocardiogram (ECG), blood pressure (BP), heart rate (HR), and pulse oxygen saturation (SpO₂) was commenced. Vital signs were documented at 5-minute intervals throughout the procedure until the patient exited the operating room. A peripheral intravenous access was established in the non-operative limb, and intravenous fluids were administered as appropriate.

### Anesthesia

Both groups of patients underwent standard general anesthesia. Induction of general anesthesia was performed with intravenous fentanyl (3 μg/kg), propofol (2.5 mg/kg), and cisatracurium besylate (0.1 mg/kg). After disappearance of the patient’s eyelash reflex, face-mask controlled ventilation was performed for 3 minutes. Subsequently, a LMA was inserted, and the patient was connected to an anesthesia machine for controlled mechanical ventilation. Ventilators settings were: tidal volume 8–10 ml/kg, respiratory rate 15–20 breaths/min. Anesthesia was maintained intraoperatively with 2%–3% sevoflurane in oxygen/air and intravenous remifentanil. Supplemental intravenous fentanyl was titrated according to hemodynamic responses and changes in vital signs. At the end of surgery, the LMA was removed after adequate recovery of spontaneous ventilation and the return of protective airway reflexes.

After the operation, the patients were transferred to the post-anesthesia care unit (PACU). In the PACU, the patients’ pain scores were evaluated regularly using a visual analog scale (VAS). When the VAS score is greater than or equal to 4, a single intravenous dose of tramadol 2 mg/kg is administered for analgesia. In the ward, oral acetaminophen is used as the primary analgesic measure.

### Ultrasound-guided FICB

After the LMA was inserted, FICB was performed under ultrasound guidance. The patient was placed in the supine position, and the inguinal region was fully exposed, routinely disinfected, and draped. The ultrasound probe was positioned at the lateral one-third of the line connecting the anterior superior iliac spine and the pubic symphysis [8]. After visualizing the anterior superior iliac spine, the probe was slid medially to identify the “bow-tie sign” formed by the fascias of the sartorius muscle and the internal oblique muscle. The in-plane technique was used for needle insertion. When the needle tip was clearly visible reaching the fascia iliac compartment on ultrasound and no blood was aspirated, 0.25% ropivacaine (0.5 mL/kg) was slowly injected.

### Observation indicators

The mean arterial pressure (MAP) and HR of the two groups of patients at various time points (including key time points such as before anesthesia induction, after induction, at the time of LMA insertion, at skin incision, and at the end of the operation) were recorded and compared. The aim was to evaluate the effect of ultrasound-guided FICB combined with LMA general anesthesia on the intraoperative hemodynamics of patients with OI. The duration of the operation, recovery time, and the amount of postoperative opioid use of the two groups of patients were observed and recorded to evaluate the effect of ultrasound-guided FICB on the surgical process and postoperative recovery, especially its effect on reducing the amount of opioid use. The levels of serum C-reactive protein (CRP) in the two groups of patients 6 hours after surgery were detected and compared to evaluate the effect of ultrasound-guided FICB on the postoperative inflammatory response. As an acute-phase reactive protein, the change in CRP level can reflect the degree of the body’s inflammatory response. The incidence of postoperative nausea and vomiting (PONV) in the two groups of patients, as well as the VAS scores at 2, 4, and 12 hours after surgery, were recorded and compared to comprehensively evaluate the analgesic effect and adverse reactions of ultrasound-guided FICB combined with LMA general anesthesia in the femoral shaft fracture surgery of OI patients.

### Statistics

Graphpad Prism (version 8.0.1) statistical software was employed for data analysis. Measurement data for normal distribution were presented as mean ± standard deviation (M ± SD). Independent-samples t-tests were conducted for inter-group comparisons of continuous variables (Welch correction where necessary). Otherwise, data were presented as median (upper and lower quartiles), and Mann-Whitney nonparametric tests were conducted for inter-group comparisons. Categorical data were expressed as percentages and analyzed using the chi-square (*χ²*) test. For repeated-measurement data, such as MAP and HR at different time points, a repeated-measures analysis of variance (ANOVA) was performed to assess the interaction between time and group factors. A two-sided *P* < 0.05 was considered statistically significant.

Propensity Score Matching (PSM) was utilized to control for confounding factors, and a competing risk model was established for analysis. Taking the group (Control group vs. FICB group) as the dependent variable and five baseline characteristics, namely Age, OI type, gender, ASA classification, and BMI, as covariates, logistic regression was employed to compute each patient’s propensity score. The 1:1 nearest neighbor matching method was adopted with a matching tolerance of 0.2 standardized mean difference (SMD). Stratified matching based on gender, ASA classification, and OI subtype guaranteed balanced sample sizes between the control group and the FICB group within each stratum.

## Results

Upon screening, a total of 126 eligible children were enrolled for the study. Among them, 70 were allocated to the Control group, and 56 were assigned to the FICB group. Through 1:1 nearest neighbor matching, a total of 98 patients were ultimately enrolled (49 in each group), with a successful matching rate of 87.5% (49/56).

Both before and after PSM, no statistically significant disparities were detected in baseline characteristics, encompassing age, BMI, gender, ASA classification, and OI type, between the two groups (Table 1).

**Table 1:** Baseline Data of Patients.

	Before PSM		*P*	After PSM		*P*
	Control group (n = 70)	FICB group (n = 56)		Control group (n = 49)	FICB group (n = 49)	
Age, years	8.84 ± 2.33	8.75 ± 2.23	0.820	8.67 ± 2.30	8.90 ± 2.30	0.658
BMI, kg/m^2^	16.24 ± 0.46	16.16 ± 0.53	0.359	16.25 ± 0.45	16.17 ± 0.46	0.170
Gender, n (%)			0.936			1.000
Male	37	30		25	25	
Female	33	26		24	24	
ASA classification, I/II	49/21	45/11	0.262	38/11	38/11	1.000
OI type, I/II/III/IV	29/0/20/21	25/0/12/19	0.653	21/11/17	21/11/17	1.000

Note: FICB, fascia iliaca compartment block; BMI, Body Mass Index; ASA, American Society of Anesthesiologists; OI, Osteogenesis Imperfecta; PSM, Propensity Score Matching.

**Fig 1:**
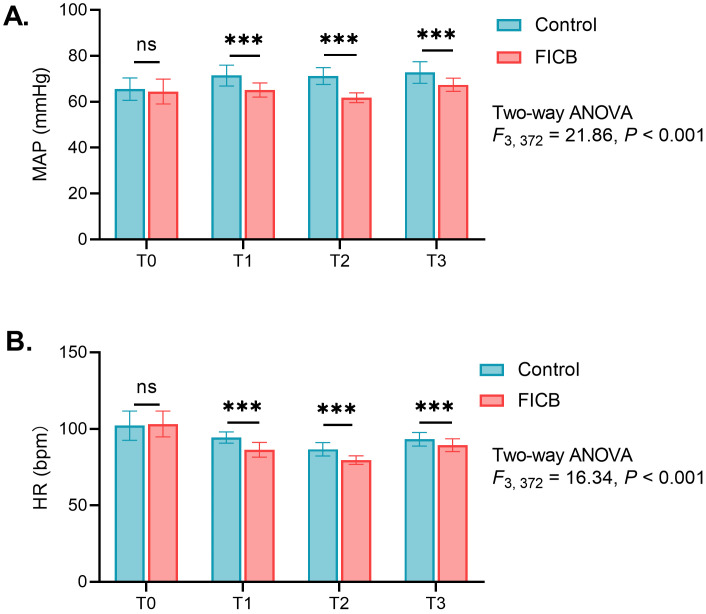
The changes in MAP and HR during surgery in the two groups. T0: before anesthesia; T1: at skin incision; T2: 20 minutes after surgery onset; T3: at the end of surgery. (A) A significant time × group interaction was observed for MAP changes during the procedure. No differences in MAP were observed between groups at T0. From T1 to T3, MAP values in the FICB group were significantly lower than those in the Control group. (B) A significant time × group interaction was also found for HR changes. HR did not differ between groups at baseline, but from T1 to T3, HR in the FICB group was consistently lower compared to the Control group. Data are presented as mean ± SD; ***P < 0.001. MAP, mean arterial pressure; HR, heart rate; FICB, fascia iliaca compartment block; ANOVA, analysis of variance.

A significant time × group interaction was observed for both the changes of MAP (*F*_3, 372_ = 21.86, *P* < 0.001, Fig 1A) and HR (*F*_3, 372_ = 16.34, *P* < 0.001, Fig 1B) during the operation between the two groups. Post-hoc analysis showed that there was no significant difference in MAP and HR between the groups before the induction of anesthesia (both *P* > 0.05). At the time of skin incision, 20 minutes after the start of surgery, and at the end of the procedure, the MAP and HR in the FICB group were significantly lower than those in the Control group (all *P* < 0.001).

**Fig 2:**
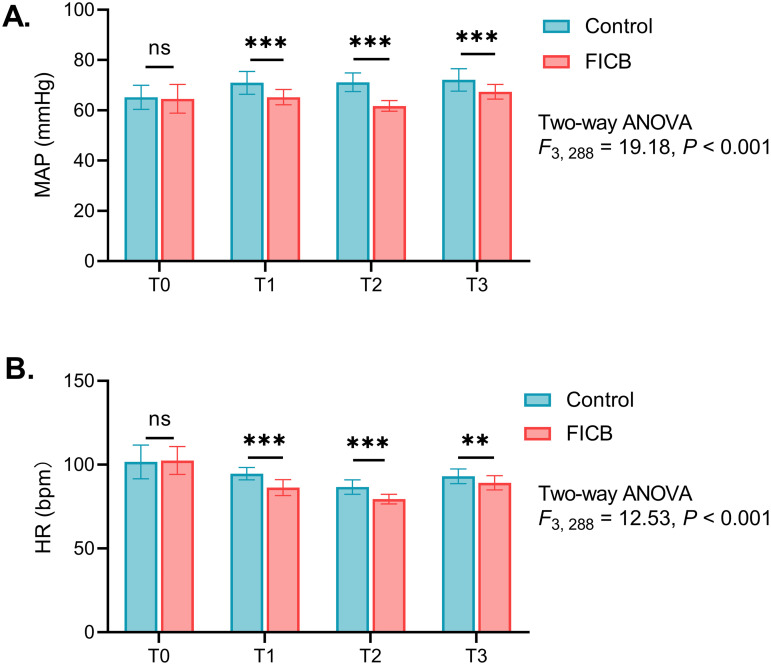
The changes in MAP and HR during surgery in the two groups after PSM. T0: before anesthesia; T1: at skin incision; T2: 20 minutes after surgery onset; T3: at the end of surgery. (A) A significant time × group interaction was observed for MAP changes during the procedure. No differences in MAP were observed between groups at T0. From T1 to T3, MAP values in the FICB group were significantly lower than those in the Control group. (B) A significant time × group interaction was also found for HR changes. HR did not differ between groups at baseline, but from T1 to T3, HR in the FICB group was consistently lower compared to the Control group. Data are presented as mean ± SD; **P < 0.01, ***P < 0.001. MAP, mean arterial pressure; HR, heart rate; FICB, fascia iliaca compartment block; ANOVA, analysis of variance.

After PSM, a significant time × group interaction was observed for both the changes of MAP (*F*_3, 288_ = 19.18, *P* < 0.001, Fig 2A) and HR (*F*_3, 288_ = 12.53, *P* < 0.001, Fig 2B) during the operation between the two groups. Post-hoc analysis showed that there was no significant difference in MAP and HR between the groups before the induction of anesthesia (both *P* > 0.05). At the time of skin incision, and 20 minutes after the start of surgery, the MAP and HR in the FICB group were significantly lower than those in the Control group (all *P* < 0.001). At the end of the procedure, the MAP (*P* < 0.001) and HR (*P* = 0.005) in the FICB group were also significantly lower than those in the Control group.

**Fig 3:**
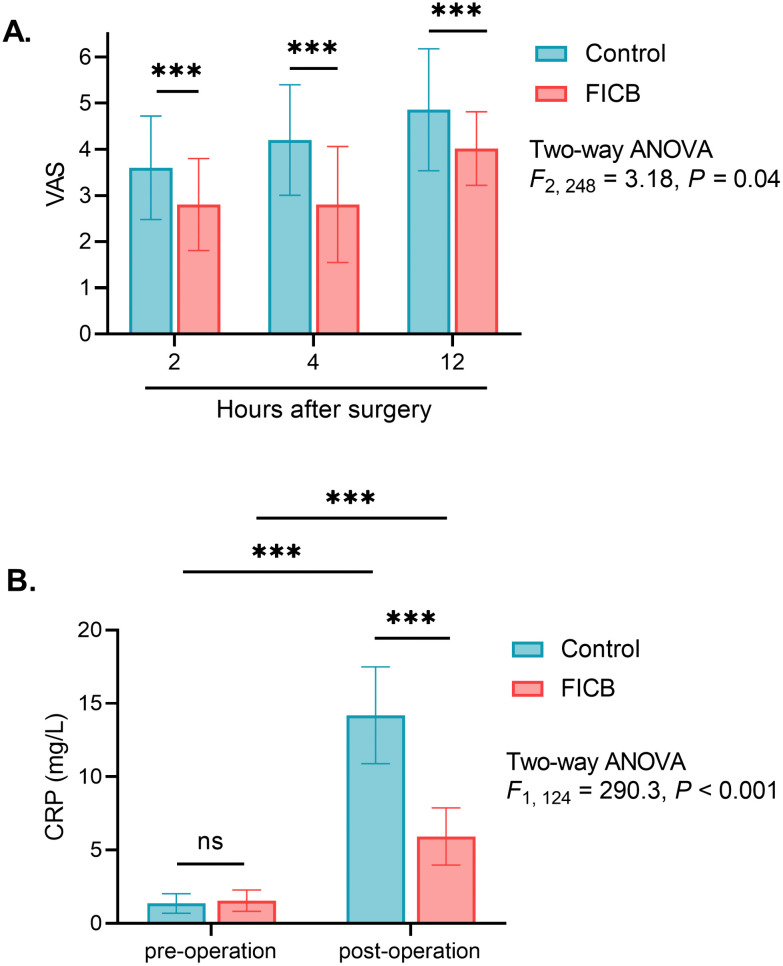
The changes in VAS scores and serum CRP levels in the two groups of children following surgery. (A) A significant time × group interaction was observed for postoperative VAS scores. At 2, 4, and 12 hours after surgery, VAS scores in the FICB group were significantly lower than those in the Control group. (B) A significant time × group interaction was also found for postoperative serum CRP levels. Both groups exhibited a marked increase in CRP at 6 hours post-surgery, but the rise was significantly lower in the FICB group compared to the Control group. Data are presented as mean ± SD; ***P < 0.001. VAS, Visual Analogue Scale; CRP, C-reactive protein; FICB, fascia iliaca compartment block; ANOVA, analysis of variance.

There was a significant time × group interaction in the changes of VAS scores after surgery between the two groups of children (*F*_2, 248_ = 3.18, *P* = 0.04, Fig 3A). Post-hoc analysis revealed that the VAS scores in the FICB group were significantly lower than those in the Control group at 2, 4, and 12 hours postoperatively (all *P* < 0.001). A significant time × group interaction was also observed in the changes of serum CRP levels between the two groups (*F*_1, 124_ = 290.3, *P* < 0.001, Fig 3B). Post-hoc analysis indicated that serum CRP levels in both groups were significantly elevated after surgery compared to baseline (all *P* < 0.001), whereas the FICB group exhibited significantly lower CRP levels than the Control group following surgery (*P* < 0.001).

**Fig 4:**
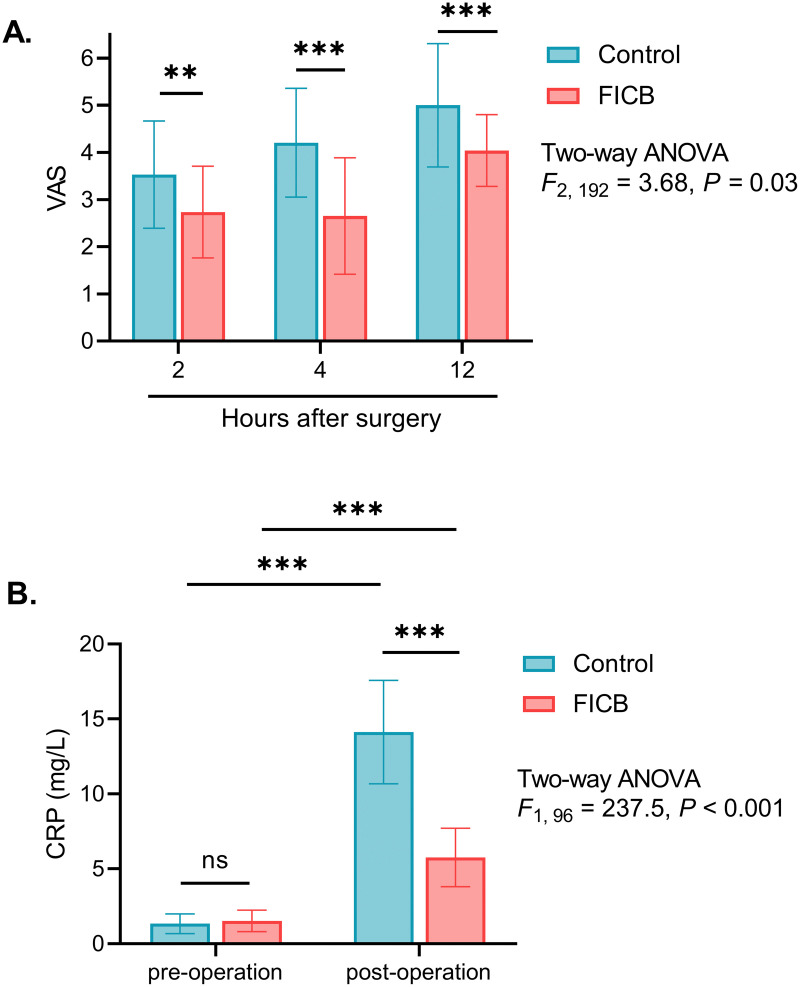
The changes in VAS scores and serum CRP levels in the two groups of children following surgery after PSM. (A) A significant time × group interaction was observed for postoperative VAS scores. At 2, 4, and 12 hours after surgery, VAS scores in the FICB group were significantly lower than those in the Control group. (B) A significant time × group interaction was also found for postoperative serum CRP levels. Both groups exhibited a marked increase in CRP at 6 hours post-surgery, but the rise was significantly lower in the FICB group compared to the Control group. Data are presented as mean ± SD; ***P < 0.001. VAS, Visual Analogue Scale; CRP, C-reactive protein; FICB, fascia iliaca compartment block; ANOVA, analysis of variance.

After PSM, there was a significant time × group interaction in the changes of VAS scores after surgery between the two groups of children (*F*_2, 192_ = 3.68, *P* = 0.03, Fig 4A). Post-hoc analysis revealed that the VAS scores in the FICB group were significantly lower than those in the Control group at 2 (*P* < 0.01), 4 (*P* < 0.001), and 12 (*P* < 0.001) hours postoperatively. A significant time × group interaction was also observed in the changes of serum CRP levels between the two groups (*F*_1, 96_ = 237.5, *P* < 0.001, Fig 4B). Post-hoc analysis indicated that serum CRP levels in both groups were significantly elevated after surgery compared to baseline (all *P* < 0.001), whereas the FICB group exhibited significantly lower CRP levels than the Control group following surgery (*P* < 0.001).

Compared with the Control group, the FICB group exhibited a significantly shorter postoperative recovery time both before and after PSM (both *P* < 0.001). Intraoperative consumption of fentanyl and remifentanil was also lower in the FICB group than in the Control group both before and after PSM (all *P* < 0.001). After adjustment for body weight, significant differences remained in the doses of fentanyl and remifentanil per kilogram of body weight between the two groups both before and after PSM (all *P* < 0.001). The incidence of PONV was significantly lower in the FICB group than in the Control group (*P* = 0.026). However, the differences between the two groups after PSM were not significant (*P* = 0.091) (Table 2). No complications related to FICB were observed during this study.

**Table 2:** Comparison of recovery time, opioid dosage, and PONV between the two groups.

	Before PSM		*P*	After PSM		*P*
	Control group (n = 70)	FICB group (n = 56)		Control group (n = 49)	FICB group (n = 49)	
Recovery time (min)	16.9 ± 2.4	12.2 ± 2.1	<0.001	17.0 ± 2.5	12.2 ± 2.1	<0.001
Fentanyl (μg)	203.4 ± 10.3	139.6 ± 14.6	<0.001*	204.1 ± 11.1	140.0 ± 14.4	<0.001
Remifentanil (μg)	250.5 ± 11.1	197.0 ± 11.3	<0.001	251.1 ± 12.2	196.72 ± 11.3	<0.001
Fentanyl/weight (μg/kg)	7.2 (5.9, 8.0)	5.4 (4.5, 6.0)	<0.001	7.3 ± 1.6	5.4 ± 1.4	<0.001
Remifentanil/weight (μg/kg)	8.9 ± 1.9	7.6 ± 1.7	<0.001	8.9 ± 1.9	7.6 ± 1.8	<0.001
PONV, n (%)			0.026			0.091
Yes	11 (15.71)	2 (3.57)		8 (16.33)	2 (4.08)	
No	59 (84.29)	54 (96.43)		41(83.67)	47(95.92)	

Note: FICB, fascia iliaca compartment block; PONV, postoperative nausea and vomiting; PSM, Propensity Score Matching. * Welch correction. Measurement data for normal distribution were presented as mean ± standard deviation. Otherwise, data were presented as median (upper and lower quartiles).

## Discussion

This research presents a series of crucial observations concerning the utilization of ultrasound-guided FICB in children with OI who are undergoing femoral shaft fracture surgery. The results indicate that the combination of FICB and LMA general anesthesia is feasible and may yield potential advantages, such as a reduction in postoperative pain scores, opioid consumption, and attenuation of the inflammatory response. Additionally, the incidence of PONV was lower in the FICB group, with no other adverse events reported. This study offers valuable preliminary evidence that supports the potential of FICB in this challenging patient cohort.

Notably, despite the retrospective nature of this study, it possesses substantial value for pediatric populations afflicted with this rare and therapeutically challenging condition, preliminarily indicating the feasibility of FICB in reducing pain scores and diminishing opioid utilization. These findings are in line with the pressing clinical requirements of children with OI, specifically the demand for regional analgesic strategies that effectively alleviate opioid dependence and its associated adverse effects. Although the current results are clinically relevant, further prospective controlled trials are necessary to validate the precise efficacy and long-term impact of FICB in this particular population.

The theoretical rationale for investigating FICB in children with OI is multifaceted. OI is a rare genetic disorder characterized by bone fragility, often necessitating multiple surgical interventions throughout childhood [1]. These children are particularly vulnerable to the adverse effects of systemic analgesics, especially opioids, which can cause respiratory depression, constipation, and prolonged sedation—complications that may be further exacerbated by the underlying skeletal and pulmonary issues in OI [2]. Regional analgesic techniques offer a compelling alternative by providing targeted pain relief, thereby potentially reducing the reliance on systemic opioids and their associated side effects. This addresses a significant unmet clinical need in this population, where optimizing pain control while minimizing risks is paramount [5]. The potential advantages of regional analgesic techniques, including improved perioperative comfort, earlier mobilization, and reduced opioid exposure, align with the broader goal of enhancing the quality of perioperative care in children with OI.

FICB is a technique that achieves analgesia in the surgical area of the lower extremities by delivering local anesthetic agents into the iliac fascia compartment. Due to its significant analgesic effect and relatively low incidence of adverse effects, this technique has been widely adopted in surgeries for lower extremity fractures [9]. FICB can effectively block the conduction of the lateral femoral cutaneous nerve, the obturator nerve, and the femoral nerve, which are branches of the lumbar plexus. These nerve branches are widely distributed in the deep layers of the thigh skin, skeletal muscle, and periosteum [10]. By blocking them, FICB can precisely and efficiently suppress nociceptive signaling from surgical stimulation, thereby significantly reducing or even eliminating pain in the surgical area [9,11].

This study applied FICB in the internal fixation surgery of femoral fractures in children with OI, further inferring the efficacy and safety of FICB in specific surgical settings [12,13]. Compared with conventional anesthesia techniques, ultrasound-guided FICB combined with LMA general anesthesia enables more precise targeting of the surgical site, reduces systemic anesthetic requirements, and thereby minimizes drug-related adverse effects on pediatric physiological systems [9,11]. This multimodal anesthetic approach—integrating regional and general anesthesia—not only enhances intraoperative anesthetic quality but also facilitates rapid postoperative recovery in pediatric patients. These findings align with previous evidence demonstrating that multimodal analgesia strategies are superior to single-modality approaches in controlling postoperative pain and reducing opioid consumption [14–16].

The anesthesia process for pediatric patients is inherently complex and presents distinct clinical challenges [14,15]. Due to incomplete physiological maturation, children exhibit significant differences from adults in metabolic capacity and pharmacological responses to anesthetic agents. Consequently, anesthetic regimens for pediatric patients require meticulous design and individualized adjustments to ensure both safety and efficacy [14,15]. These considerations become even more critical when managing patients with OI, a genetic disorder characterized by brittle bones and increased susceptibility to fractures. This condition heightens vulnerability to surgical stress and external stimuli, particularly during the intraoperative and postoperative phases. To mitigate perioperative stress responses and minimize fracture risk, anesthesiologists must carefully optimize drug selection, dosage titrations, and monitoring strategies. A comprehensive and tailored anesthetic approach not only supports hemodynamic stability and patient comfort but also significantly contributes to successful surgical outcomes and uneventful recovery [16,17].

Hemodynamic stability is a critical component of perioperative anesthesia management and plays a pivotal role in determining the prognosis of pediatric patients with OI [16,17]. In this study, FICB was integrated into clinical anesthesia protocols to achieve a more stable intraoperative hemodynamic profile in children with OI, aligning with findings from previous studies [18]. At key intraoperative time points, the FICB group exhibited significantly smaller fluctuations in MAP and greater HR stability compared to the Control group, demonstrating the effectiveness of FICB in maintaining circulatory homeostasis. Furthermore, HR levels in both groups were elevated prior to anesthesia induction relative to measurements taken at the onset of surgery, 20 minutes intraoperatively, and at the end of the procedure. This pattern likely reflects sympathetic nervous system activation due to preoperative psychological stress in pediatric patients [19,20], potentially mediated by hyperactivity of the locus coeruleus-noradrenergic system [21]. In contrast, blood pressure remained relatively unchanged, attributable to high vascular compliance and effective maintenance of intravascular volume [22]. The judicious use of anesthetic techniques and pharmacological agents may thus enable precise modulation of the perioperative stress response through combined central receptor modulation and peripheral nerve blockade [23].

This study indicates that VAS scores in the FICB group at 2, 4, and 12 hours postoperatively were significantly lower than those in the Control group. These findings further support the notion that precise blockade of the pain conduction pathway within the surgical field not only effectively suppresses intraoperative nociceptive responses but also provides sustained and effective postoperative analgesia in pediatric patients [24]. Notably, pain scores in both groups exhibited an increasing trend over time, with higher values observed at 12 hours compared to those at 2 and 4 hours postoperatively. This pattern may be attributed to the pharmacokinetic properties of 0.25% ropivacaine used in the FICB group: as it diffuses through the iliofascial space, it provides continuous sensory nerve blockade for approximately 2–8 hours after administration, thereby delaying the rebound of pain following systemic analgesic clearance. By 12 hours post-surgery, although the local concentration of ropivacaine likely falls below the effective analgesic threshold, early suppression of nociceptive signaling may have attenuated central sensitization in spinal dorsal horn neurons, resulting in a significantly attenuated increase in pain intensity relative to the Control group [25].

The application of ultrasound-guided FICB in pediatric femoral shaft fracture surgery provides effective analgesia and reduces the risk of respiratory depression associated with excessive opioid use, a critical advantage in pediatric patients [26]. Furthermore, this technique lowers the incidence of postoperative adverse events, improves postoperative comfort, and facilitates earlier recovery in children [12,13,25]. The findings of this study support the broader adoption of ultrasound-guided FICB combined with LMA general anesthesia for pediatric femoral shaft fracture surgery. However, it is important to recognize that age, body weight, and clinical severity may influence individual responses to anesthesia [12,13]. Future studies should employ more refined subgroup analyses to identify the optimal anesthetic strategy for specific pediatric populations.

In terms of safety, the incidence of PONV in the FICB group was significantly lower than in the Control group, a difference likely attributable to reduced intraoperative use of opioid-based general anesthetics [27]. Opioids induce gastrointestinal discomfort by activating μ-opioid receptors located on presynaptic nerve terminals in the myenteric plexus, thereby inhibiting gastrointestinal motility [28]. Additionally, activation of opioid receptors in the central nervous system can directly trigger PONV [29]. A substantial body of evidence indicates that intraoperative opioid overuse not only increases the risk of PONV but also delays patient recovery, findings that align closely with those of the present study [27]. Therefore, appropriate regulation of opioid dosage is crucial for enhancing postoperative safety and minimizing complications.

It is particularly noteworthy that the serum CRP level in the FICB group was lower than that in the Control group six hours after surgery, indicating that the sustained analgesic effect of FICB may be mediated through suppression of peripheral inflammatory mediator release. Both surgical trauma and anesthesia can activate the immune system to varying degrees, leading to the release of pro-inflammatory cytokines such as interleukin-6 (IL-6), which in turn stimulate the liver to synthesize and release CRP, thereby elevating systemic CRP levels [30–33]. Consequently, CRP levels in both groups of children were elevated postoperatively compared to preoperative values. Given that CRP levels are closely associated with postoperative complications such as infection and organ dysfunction [30,31], this finding underscores the clinical relevance of modulating the inflammatory response. This study evaluated the impact of FICB combined with LMA general anesthesia on postoperative inflammatory response, compared with LMA general anesthesia alone. The results demonstrated that the FICB group exhibited significantly lower serum CRP levels six hours after surgery than the control group. This reduction may be attributed to the ability of FICB to more effectively attenuate the surgical stress response, thereby decreasing the release of pro-inflammatory hormones such as catecholamines and cortisol, which subsequently dampens systemic inflammation [18,34]. Furthermore, emerging evidence suggests that peripheral nerve blocks and local anesthetics may directly modulate immune cell function, potentially contributing to the observed anti-inflammatory effects [34–36].

This study also presents certain limitations. As a single-center retrospective cohort study, it is susceptible to selection bias and confounding by indication. This study employed PSM to reduce bias, yet it failed to completely eliminate bias. The generalizability of results could be improved through future multi-center prospective studies with larger sample sizes, which could further validate these findings across various clinical contexts. The incomplete collection of certain clinically relevant variables, such as fracture severity, surgical complexity, baseline pain sensitivity, and postoperative opioid use, restricts the ability to fully account for potential confounders. Despite our attempts to balance known factors through study design and statistical adjustment, unmeasured heterogeneity may still affect the interpretation of the outcomes. Future research would be well-served by standardized data collection protocols to ensure more comprehensive covariate identification. In terms of assessing the inflammatory response, CRP measurements were collected at a single time point (6 hours postoperatively), limiting our ability to characterize the long-term inflammatory trajectory following FICB. Although this time point was selected based on prior evidence indicating early CRP elevation as a marker of acute inflammatory activation, extended monitoring over 24–72 hours could provide greater insight into the duration and magnitude of the anti-inflammatory effects. Therefore, we recommend prolonged biomarker surveillance in future trials. Finally, due to sample size limitations, the current analysis did not include stratified assessments by key subgroups. Exploratory subgroup analyses in larger cohorts may help identify differential treatment effects and optimize patient selection criteria for regional anesthesia techniques.

## Conclusion

In pediatric patients with OI undergoing femoral shaft fracture surgery, ultrasound-guided FICB has the potential to enhance intraoperative hemodynamic stability, alleviate postoperative pain, and decrease the incidence of adverse reactions, thereby facilitating the implementation of optimized multimodal analgesia.
