# Supplementary material for: Fascia iliaca compartment block for surgical analgesia in children with osteogenesis imperfecta: A retrospective cohort study
Source: PLoS One. 2026 Jul 2;21(7):e0353186. doi: 10.1371/journal.pone.0353186 (PMC13327246; doi:10.1371/journal.pone.0353186)
Supplement: Attachment — Submitted filename: Response to Reviewers.pdf [file pone.0353186.s002.pdf]

PONE-D-25-58723

Fascia Iliaca Compartment Block for Surgical Analgesia in Children with Osteogenesis Imperfecta: A Retrospective Cohort Study

Dear editor Robert Jeenchen Chen,

We hope this message finds you well! And we wish you a happy Chinese Spring Festival!

We sincerely appreciate your review and guidance on our manuscript, as well as that of the two reviewers. We fully recognize that your expertise can significantly enhance the quality of the manuscript. We understand that these are revisable aspects, and we believe that with some revisions, our manuscript can reach a high-quality standard suitable for publication.

Below is our point-by-point response to the comments, along with corresponding revisions. Highlight the modified content in red in the manuscript.

Please do not hesitate to contact us if you encounter any questions during the revision process.

Best regards,

Xueli Jiang

Journal Requirements:

When submitting your revision, we need you to address these additional requirements.

1. Please ensure that your manuscript meets PLOS ONE's style requirements, including those for file naming. The PLOS ONE style templates can be found at https://journals.plos.org/plosone/s/file?id=wjVg/PLOSOne_formatting_sample_main_body.pdf and https://journals.plos.org/plosone/s/file?id=ba62/PLOSOne_formatting_sample_title_authors_affiliations.pdf.

Response: We have revised the article to ensure that it conforms to the journal format requirements.

2. Please provide additional details regarding participant consent. In the ethics statement in the Methods and online submission information, please ensure that you have specified (1) whether consent was informed and (2) what type you obtained (for instance, written or verbal, and if verbal, how it was documented and witnessed). If your study included minors, state whether you obtained consent from parents or guardians. If the need for consent was waived by the ethics committee, please include this information.

If you are reporting a retrospective study of medical records or archived samples, please ensure that you have discussed whether all data were fully anonymized before you accessed them and/or whether the IRB or ethics committee waived the requirement for informed consent. If patients provided informed written consent to have data from their medical records used in research, please include this information.

Response: This study is a retrospective study that has undergone ethical review and was exempt from informed consent.

Revision: Add a statement in the methods section that informed consent was waived (lines ...).

3. Thank you for stating in your Funding Statement:

"This study was financially supported by the Natural Science Foundation of Jiangsu Province (BK20241800)"

Please provide an amended statement that declares *all* the funding or sources of support (whether external or internal to your organization) received during this study, as detailed online in our guide for authors at http://journals.plos.org/plosone/s/submit-now. Please also include the statement "There was no additional external funding received for this study." in your updated Funding Statement.

Please include your amended Funding Statement within your cover letter. We will change the online submission form on your behalf.

Response: We have supplemented the Funding Statement as required and included the

statement in the cover letter.

4. Thank you for stating the following financial disclosure:

"This study was financially supported by the Natural Science Foundation of Jiangsu Province (BK20241800)"

Please state what role the funders took in the study. If the funders had no role, please state: "The funders had no role in study design, data collection and analysis, decision to publish, or preparation of the manuscript."

If this statement is not correct you must amend it as needed.

Please include this amended Role of Funder statement in your cover letter; we will change the online submission form on your behalf.

Response: We have supplemented the Funding Statement as required and included the statement in the cover letter.

5. We note that your Data Availability Statement is currently as follows: [All relevant data are within the manuscript and its Supporting Information files.]

Please confirm at this time whether or not your submission contains all raw data required to replicate the results of your study. Authors must share the "minimal data set" for their submission. PLOS defines the minimal data set to consist of the data required to replicate all study findings reported in the article, as well as related metadata and methods (https://journals.plos.org/plosone/s/data-availability#loc-minimal-data-set-definition).

For example, authors should submit the following data:

- The values behind the means, standard deviations and other measures reported;
- The values used to build graphs;
- The points extracted from images for analysis.

Authors do not need to submit their entire data set if only a portion of the data was used in the reported study.

If our submission does not contain these data, please either upload them as Supporting

Information files or deposit them to a stable, public repository and provide us with the relevant URLs, DOIs, or accession numbers. For a list of recommended repositories, please see https://journals.plos.org/plosone/s/recommended-repositories.

If there are ethical or legal restrictions on sharing a de-identified data set, please explain them in detail (e.g., data contain potentially sensitive information, data are owned by a third-party organization, etc.) and who has imposed them (e.g., an ethics committee). Please also provide contact information for a data access committee, ethics committee, or other institutional body to which data requests may be sent. If data are owned by a third party, please indicate how others may request data access.

Response: All statistical results are included in our manuscript. Additionally, our raw data are uploaded as Supporting Information files during the revision process.

To Reviewer #1,

We hope this message finds you well! And we wish you a happy Chinese Spring Festival!

We sincerely appreciate your review and guidance on our manuscript. We fully recognize that your expertise can significantly enhance the quality of the manuscript. We understand that these are revisable aspects, and we believe that with some revisions, our manuscript can reach a high-quality standard suitable for publication.

Below is our point-by-point response to the comments, along with corresponding revisions. We have put together some of the relevant comments to respond to.

Please do not hesitate to contact us if you encounter any questions during the revision process.

Best regards,

Corresponding author (XJ)

1. The manuscript would benefit from a clearer explanation of how patients were selected for receiving FICB versus standard analgesia.

2. It remains unclear whether the decision to perform FICB was influenced by patient characteristics, surgeon preference, anesthesiologist experience, or temporal changes in practice, which introduces a potential selection bias.

Response: Thank you for your review and guidance. This study is a retrospective study designed to compare the efficacy of FICB in the management of OI patients undergoing surgery. Given the rarity of OI surgical cases in clinical practice and the extended observation period, it was challenging to design a fully randomized prospective study. FCIB is a well-established clinical technique. Prior to surgery, anesthesiologists (rather than investigators) determine whether a patient is suitable for FICB analgesia based on comprehensive factors such as the patient's specific condition, and thoroughly communicate with the patient and their family. The final choice of analgesic method is entirely determined by the patient and their family. All clinical data for the patients in this study were retrospectively collected from medical records, and the anesthesiologists' decision-making process was based on clinical

experience and the patient's actual condition, without any intervention by the investigators.

Regarding potential selection bias, although anesthesiologists' decisions may be influenced by multiple factors, we implemented a series of measures during the data analysis phase to minimize the impact of such bias. We compared patients' baseline characteristics and surgical-related information to ensure comparability between groups, thereby enabling an accurate assessment of the efficacy difference between FICB and standard analgesia in OI patients undergoing surgery. Additionally, we acknowledge the inherent limitations of retrospective studies, which cannot completely eliminate all potential selection biases. However, in the context of research on rare diseases, retrospective studies can provide valuable clinical evidence. In the future, we plan to conduct prospective randomized controlled trials to further clarify the optimal application and efficacy of FICB in OI patients undergoing surgery, employing more rigorous study designs to avoid potential bias issues.

Revision: Add a detailed description of 'Patient Selection/Grouping Criteria' in the 'Methods' section. Expand the content in the 'Discussion/Limitations' section to candidly discuss the inherent selection bias (Selection Bias) and indication confounding (Confounding by Indication) issues in retrospective studies.

3. Confounding factors (e.g., fracture severity, surgical complexity, OI subtype, baseline pain sensitivity) are not sufficiently explored.

4. Please clarify patient selection or expand the limitations section to explicitly discuss selection bias, confounding by indication, and the absence of randomization.

5. OI encompasses a wide spectrum of severity (types I–V and beyond), which may significantly affect pain perception, fracture burden, and postoperative recovery. It is unclear whether disease severity or subtype was comparable between groups. If available, provide data on OI type, clinical severity, or surrogate markers (e.g., fracture history, bisphosphonate therapy). If not available, this should be explicitly stated as a limitation.

Response: Thank you for your review and guidance. We recognize that confounding

factors play a crucial role in the study's results. Due to the nature of this retrospective study and the limited data sources from medical records, comprehensive exploration of all confounding factors was a challenge. Nevertheless, we can confirm that we did make efforts to collect and analyze information on these factors as much as possible. The OI type was relatively easy to obtain. We have supplemented it into the baseline information, performed inter-group comparisons, and verified the inter-group balance. However, due to insufficiently standardized and detailed documentation in medical records regarding fracture severity and surgical complexity, we have difficulty obtaining comprehensive and accurate data. This indeed represents a significant limitation in the current study. In subsequent research, we will focus on improving data collection methods and standards to ensure more thorough acquisition of this critical information.

Regarding baseline pain sensitivity, this is a challenging metric to precisely quantify, and there is a lack of objective documentation in medical records. We recognize that this factor may influence the study outcomes, but due to the retrospective nature of this study and the limitations of available data, we were unable to thoroughly investigate it. In future prospective studies, we will employ appropriate assessment tools and methods to accurately evaluate and analyze patients' baseline pain sensitivity.

Regarding the comparability of disease severity or subtypes between the two groups, although we have supplemented the information on OI types and conducted intergroup balance validation, we are also aware that this may not fully represent the severity of the disease. If there are additional data on clinical severity or surrogate markers, such as fracture history or bisphosphonate therapy, we will further collect and analyze them, and supplement the results in the study. If such data cannot be obtained, we will explicitly state this as a limitation of the study in the manuscript to ensure the scientific rigor and integrity of the research. Simultaneously, we will strive to improve the data collection system in future studies to minimize limitations caused by data gaps, thereby providing more reliable clinical evidence for the application of FICB in OI patients during surgery.

Revision: The comparison of OI typing was added in the results section and Table 1. In the limitations section, the absence of certain key baseline data and its implications were stated.

6. The timing of the block (preoperative vs postoperative), local anesthetic concentration and volume, and use of ultrasound guidance should be described in greater detail. It is unclear whether intraoperative and postoperative systemic analgesic regimens were standardized between groups.

Provide a clearer description of the analgesic protocol and clarify whether other analgesics (e.g., acetaminophen, NSAIDs, opioids) were administered in a standardized manner.

Response: We sincerely appreciate your valuable suggestions. The detailed procedure of ultrasound-guided FICB, including the block time point, local anesthetic concentration, and dosage, is provided in the Methods section.

As for the standardization of intraoperative and postoperative systemic analgesic regimens between groups, we understand that the lack of clarity was a concern. After a thorough review of the medical records, we found that while there was a general protocol in place, there were some variations in the actual administration of analgesics due to individual patient needs. We will clearly describe the general analgesic protocol in the manuscript and also mention the possible variations that occurred.

We will also clarify the use of other analgesics, such as acetaminophen. We have data on the frequency and dosage of these medications administered to each patient, and we will present this information in a tabular form. If the administration of these analgesics was not fully standardized, we will state this as a limitation in the study and discuss its potential impact on the results. To this end, we have added a chapter to the Method section to elaborate on this. However, due to the inability to document oral acetaminophen use in detail, the evaluation of postoperative efficacy was not considered a primary outcome in this study.

In future studies, we will ensure that all these details are carefully recorded and reported from the start to avoid any ambiguity and to provide more reliable evidence

for clinical practice. We believe that these revisions will enhance the quality and clarity of our manuscript and address your concerns effectively.

Revision: The Methods section was expanded to include the implementation of postoperative analgesia. The Limitations section included a statement regarding the potential impact of missing data on postoperative analgesia (oral analgesics) on the evaluation results.

7. Pain scores and opioid consumption are appropriate primary outcomes; however, their assessment raises several concerns. The manuscript should clarify whether validated, age-appropriate pain scales were consistently used across all patients.

8. The timing of pain assessments (e.g., PACU, first 24 hours, later postoperative period) should be clearly specified. Opioid consumption should ideally be normalized (e.g., morphine equivalents per kg) to allow meaningful comparison.

Clarify pain assessment methodology and provide justification for the chosen outcome time points.

Response: We acknowledge the importance of these concerns. The Visual Analogue Scale (VAS) was feasible and widely used in clinical practice among the 6~15 year-old population enrolled in this study. As a retrospective study, the collected clinical data were assessed using VAS scores, which could not be altered. The manuscript provided the time points for evaluation. Finally, to more effectively compare differences in analgesic efficacy, the study results included not only the total doses of fentanyl and remifentanil but also the dose adjusted per kilogram of body weight for comparison.

Revision: In the results section and Table 2, the dose adjusted per kilogram of body weight for comparison was added.

To Reviewer #2,

We hope this message finds you well! And we wish you a happy Chinese Spring Festival!

We sincerely appreciate your review and guidance on our manuscript. We fully recognize that your expertise can significantly enhance the quality of the manuscript. We understand that these are revisable aspects, and we believe that with some revisions, our manuscript can reach a high-quality standard suitable for publication.

Below is our point-by-point response to the comments, along with corresponding revisions. In addition, we have spent a great deal of time and effort on repeated self-review and revision of the manuscript to maximize its accuracy.

Please do not hesitate to contact us if you encounter any questions during the revision process.

Best regards,

Corresponding author (XJ)

The study is interesting. However, as authors themselves mentioned huge limitations of the results, the importance and significance are significantly compromised. The very nature of being retrospective and spanned over nearly six years with small number of patients, unequal groups and significant selection bias raise red flags. Why cohort study design is mentioned needs clarity.

Response: We understand your concerns regarding the limitations of our study. The retrospective nature and the long-spanning time frame of nearly six years, along with the small number of patients, unequal groups, and selection bias are indeed issues that we are well-aware of.

Regarding the cohort study design, we initially mentioned it because we aimed to observe the natural course of the disease and the outcomes of the treatment in a group of patients over time. We believe that this design was appropriate as it allowed us to collect real-world data on the pain management of OI patients undergoing surgery with FICB. However, we also recognize that the limitations associated with the retrospective design have affected the strength of our findings.

To address these concerns, we will add a more in-depth discussion in the manuscript about the limitations of our study. We will clearly state how the retrospective nature, the long-term data collection, the small sample size, the unequal groups, and the selection bias may have influenced the results. We will also discuss the potential impact of these limitations on the generalizability of our findings. Despite the limitations, our study still has some value in contributing to the existing knowledge in the field.

We believe that by being transparent about the limitations and taking steps to address them, we can enhance the credibility of our study and provide a more balanced view of the pain management outcomes in OI patients undergoing surgery with FICB.

Revision: The significance and importance of this study were emphasized in the introduction. The limitation section was revised.

It is also strange that mean values of age and BMI are almost similar. It can not be a coincidence.

Pediatric anesthesia is safe and multimodal pain therapy is a routine process. In this study ultrasound guided block is added and it is expected to improve pain. There is no new information in that context.

Response: We acknowledge your concerns about the similar mean values of age and BMI. We understand that this might seem unusual at first glance. In our study, the similarity in these values could be a result of the developmental characteristics of the disease and the selection of surgical timing by surgeons. We have only presented this result truthfully and commit to the authenticity of the data.

Regarding your comment about there being no new information with the addition of the ultrasound - guided block in the context of pediatric anesthesia and multimodal pain therapy, we would like to clarify. While pediatric anesthesia is generally safe and multimodal pain therapy is a routine process, the application of FICB in the specific population of OI patients is quite rare. OI patients have unique characteristics such as bone fragility, a history of repeated fractures, possible chronic pain, and high anesthesia risks. These factors make their pain management a special challenge.

The use of FICB in this group has the potential to reduce the consumption of opioid drugs, thereby decreasing the risks of respiratory depression and fractures. Our study is one of the few to explore this specific area, and it fills a data gap in the literature. We believe that this exploration provides valuable insights for future research and clinical practice in the pain management of OI patients.

In the manuscript, we will further strengthen the description of these points in the "Introduction" and "Discussion" sections. We will highlight the scarcity of applying FICB to this special pediatric OI population and emphasize the clinical significance of our research. We will also expand on how these unique patient characteristics make our study's findings particularly relevant and important for improving the pain management strategies for OI patients.

We sincerely appreciate your in-depth review and constructive comments. Your insights have been extremely helpful in guiding us to improve the quality of our manuscript. We are committed to making the necessary revisions to address all the concerns you have raised and to present a more comprehensive and valuable research paper.

Revision: Some data were corrected, such as the unification of decimal places and the verification of statistics. The significance and importance of this study were emphasized in the introduction and Discussion section.

It is also come as a surprise that over six years all patients were subjected to large number investigation / tests like CRP and others which are not performed routinely. What may be the reason for this?

Response: We understand your surprise regarding the large number of investigations and tests, such as CRP, conducted on all patients over the six-year period. In the case of OI patients, their condition is complex and is accompanied by various potential complications. These patients often have bone fragility, repeated fractures, and possible chronic pain, which may lead to systemic inflammation and other issues.

CRP is an important indicator of inflammation. Regularly measuring CRP can help us monitor the patient's inflammatory state, which is crucial for understanding the

patient's overall condition and guiding treatment.

This is the essential monitoring protocol for surgeons in this disease category. In a rare disease like OI, there is a lack of a large-scale standardized testing protocol. Therefore, in our clinical practice, we need to conduct a relatively comprehensive examination to obtain as much information as possible about the patient's condition. This helps us to make more accurate diagnoses and treatment decisions.

We are grateful for your careful review and this thought-provoking question. It has made us realize the importance of clearly explaining the rationale behind our testing procedures in the manuscript. We will make the necessary revisions to ensure that the manuscript is more complete and understandable.

Revision: None.

Reference citation is also not appropriate. Large number of references are not required. Reference number 8 cited in methods section is of modified radical mastectomy and has no relevance. There is verbosity and unnecessary comparison of variables while objective could have been addressed by clinical observations done objectively.

Response: We apologize for the inappropriate reference citation in the manuscript. We recognize the issue with having a large number of references and the irrelevance of Reference number 8 cited in the methods section. We will conduct a thorough review of all the references used in the manuscript.

Regarding Reference number 8, we will remove it from the methods section immediately as it is clearly of no relevance to our current study about OI patients and their treatment. We will replace it with more appropriate and related references that can better support the methods and findings presented in the manuscript.

In addition, we understand that there is verbosity and unnecessary comparison of variables in the manuscript. We will go through the text item-by-item and make the language more concise. Instead of elaborate and redundant comparisons, we will rely on objective clinical observations to address the research objectives. We will present the data and results in a more straightforward and clear manner, avoiding any unnecessary complexity.

We are committed to making these revisions to improve the quality of the manuscript. We appreciate your detailed review and these important suggestions, which have helped us identify the areas that need improvement. We will ensure that the revised manuscript has a more appropriate reference citation and a more concise and objective presentation of the research.

Revision: The whole text has been revised thoroughly and carefully, including references, sentences and logical structure.
