# Supplementary material for: Fascia iliaca compartment block for surgical analgesia in children with osteogenesis imperfecta: A retrospective cohort study
Source: PLoS One. 2026 Jul 2;21(7):e0353186. doi: 10.1371/journal.pone.0353186 (PMC13327246; doi:10.1371/journal.pone.0353186)
Supplement: Attachment — Submitted filename: Response to Reviewers 0323.pdf [file pone.0353186.s003.pdf]

PONE-D-25-58723R1

Fascia Iliaca Compartment Block for Surgical Analgesia in Children with Osteogenesis Imperfecta: A Retrospective Cohort Study

Dear editor Robert Jeenchen Chen,

We hope this message finds you well!

We sincerely appreciate your review and guidance on our manuscript, as well as that of the three reviewers. We fully recognize that your expertise can significantly enhance the quality of the manuscript. We understand that these are revisable aspects, and we believe that with some revisions, our manuscript can reach a high-quality standard suitable for publication.

Below is our point-by-point response to the comments, along with corresponding revisions. Highlight the modified content in red in the manuscript.

Please do not hesitate to contact us if you encounter any questions during the revision process.

Best regards,

Xueli Jiang

To Reviewer #1:

All comments have been addressed. The manuscript is technically sound, and the data support the conclusions.

Response: We are deeply grateful to you for your careful review and positive evaluation of our manuscript. Your confirmation that all comments have been addressed and that the manuscript is technically sound with data supporting the conclusions is extremely encouraging. We have taken this feedback to heart and are committed to maintaining this standard in the final version of the manuscript. The thoroughness of your assessment has provided us with confidence that our work meets the rigorous criteria of the journal. We have made sure to incorporate all suggested improvements and have double-checked the technical aspects and data interpretation to ensure the highest level of accuracy and validity. We believe that these efforts have strengthened the manuscript significantly, and we are thankful for your valuable input which has contributed to its refinement.

Revision: None.

To Reviewer #2:

The response is based upon large number of limitations for almost all the observations. This nullifies the purpose of research. Readers may not get an impression that are reading a compromised study.

Response: We sincerely appreciate your pointing out this critical concern, which has prompted us to thoroughly reflect on the presentation of limitations in our manuscript. We acknowledge that the initial emphasis on limitations might have overshadowed the potential value of our research, inadvertently creating an impression of a "compromised study." This was never our intention, and we deeply regret any confusion it may have caused.

First, we would like to clarify that while we have been transparent about the limitations—an essential aspect of scientific rigor—we do not view our study as inherently compromised. Instead, we believe it addresses an important gap in the literature, particularly regarding the use of FICB for surgical analgesia in a highly vulnerable and understudied population: children with OI. OI children present unique challenges in pain management due to their skeletal fragility, potential for opioid sensitivity, and the need for multiple surgeries. There is a paucity of data specifically examining regional analgesic techniques in this patient group, making even observational data valuable for guiding clinical practice.

To address your concern, we have revised the manuscript to better balance the discussion of limitations with a clearer articulation of the study's strengths and contributions. Specifically, we have:

1. Strengthened the introduction and discussion of the research gap: We have expanded on the rationale for studying FICB in OI children, emphasizing the unmet clinical need and the potential benefits of regional analgesia in this population (e.g., reducing opioid exposure and associated side effects). This provides a stronger foundation for why the study was conducted and its relevance despite its retrospective nature.

2. Reorganized the limitations section: Instead of presenting limitations upfront in a manner that might have seemed overwhelming, we have integrated a more measured

discussion of limitations within the broader context of the study's findings and their implications. We have clearly distinguished between inherent limitations of retrospective cohort studies (e.g., potential selection bias, lack of randomization) and the specific steps we took to mitigate these (e.g., detailed chart review, inclusion of a comparator group where possible, sensitivity analyses).

3. Highlighted the study's strengths: We have explicitly stated the strengths, such as the focus on a rare and clinically challenging population, the relatively large sample size for such a specific cohort, the detailed collection of perioperative analgesic data, and the demonstration of FICB's feasibility and effectiveness in terms of pain scores and opioid reduction in this context.

4. Tempered the language regarding limitations: We have revised the language to avoid overly negative framing. For example, instead of stating that a limitation "nullifies" a finding, we now discuss how it "should be considered when interpreting" the result, and we offer suggestions for future research to address these limitations.

Our goal is to present a balanced and honest account of our work, one that acknowledges its constraints while also clearly communicating the novel insights it provides. We believe these revisions will provide readers with a more accurate and nuanced understanding of the study, allowing them to appreciate its contributions to clinical knowledge in this specialized area. We hope that with these changes, the manuscript will no longer convey the impression of a "compromised study" but rather a valuable, hypothesis-generating investigation that addresses an important clinical question.

5. Fact-based expression style: During revisions, we should candidly acknowledge the insufficient evidentiary strength of research findings and strive to avoid excessive interpretation and affirmative statements. Indeed, we fully concur with the importance of presenting the evidence strength of our research findings with the utmost honesty and avoiding over-interpretation or overly assertive claims. In light of this, we have meticulously revised the discussion section to ensure that our conclusions are firmly grounded in the observed data and that we refrain from making unwarranted extrapolations. Our aim is to present the data in a manner that is both transparent and

responsible, allowing readers to form their own informed judgments about the implications of our work while acknowledging its inherent boundaries. This approach, we believe, is essential to maintaining scientific integrity and ensuring that our research contributes meaningfully to the existing body of knowledge without overstating its current scope or certainty.

Revision: We have made extensive revisions to the Discussion section, particularly the subsections on "Limitations" and the concluding paragraphs, to better balance the presentation of limitations with the study's strengths and clinical relevance. These revisions are highlighted in red in the manuscript.

Reviewer #3: The authors present a revised manuscript on the use of regional anesthesia in osteogenesis imperfecta patients. This is an interesting collection of cases and the paper is well-written. While I did not review the original manuscript, it seems that the authors have addressed the comments well.

My major concern with the manuscript is that the data are presented as if the study is designed to determine whether the block is effective or not. The study is not randomized or blinded, and patient allocation and intraoperative anesthetic were not controlled. It would therefore be more appropriate to present the results as a case series or feasibility study. This could be accomplished primarily by tempering the discussion to avoid arguing that the results support the effectiveness of the block.

Response: We sincerely appreciate your insightful observation and whole-heartedly concur with this crucial point. The retrospective, non-randomized, and non-blinded characteristics of our study, along with the lack of control over patient allocation and intraoperative anesthetics, truly prevent us from making conclusive claims about the efficacy of FICB in children with OI.

We recognize that our study was not designed to formally assess the effectiveness of the block as a randomized controlled trial would. However, while acknowledging the inherent limitations of these retrospective studies, we still recognize their significant value, albeit limited in scope.

In view of this, we have made substantial revisions to reframe the manuscript. Our primary focus is on depicting our experience with FICB in this specific patient population and exploring its potential role as part of a multimodal analgesic approach. Specifically, we have refined the language throughout the manuscript, especially in the Abstract, Results, and Discussion sections, to avoid suggesting causal efficacy. Instead of stating that "FICB is effective," we now stress that "our observations indicate that FICB might be a feasible and potentially advantageous component of perioperative pain management in OI children, meriting further investigation." We have eliminated any overly assertive claims regarding the block's effectiveness and instead spotlight the descriptive nature of our findings, the trends noted (such as decreased opioid consumption and improved pain scores in the FICB group), and their

potential implications for clinical practice and future research design. Our current objective is to present these cases as valuable clinical experience that enriches the limited body of knowledge in this area and serves as a basis for generating hypotheses and devising more rigorous prospective studies to formally evaluate the efficacy and safety of FICB in OI children. These revisions are marked in red in the manuscript.

Revision: We have comprehensively reframed the manuscript to explicitly position it as a case series/feasibility study. This includes modifying revising the Abstract to clearly state the study's purpose as describing our experience with FICB in OI children and exploring its feasibility and potential role in multimodal analgesia, and adjusting the Introduction to emphasize the paucity of data in this area and the need for such descriptive reports. Throughout the Results and Discussion section, we have rephrased statements to focus on observed trends and descriptive statistics rather than inferential claims about effectiveness. The Discussion now centers on interpreting these observations within the context of the study's limitations, highlighting the feasibility and potential benefits suggested by the cases, and emphasizing the need for future controlled trials to confirm these preliminary findings. These extensive revisions are highlighted in red in the manuscript.
