# Supplementary material for: Fascia iliaca compartment block for surgical analgesia in children with osteogenesis imperfecta: A retrospective cohort study
Source: PLoS One. 2026 Jul 2;21(7):e0353186. doi: 10.1371/journal.pone.0353186 (PMC13327246; doi:10.1371/journal.pone.0353186)
Supplement: Attachment — Submitted filename: Response to Reviewers 0424.pdf [file pone.0353186.s004.pdf]

PONE-D-25-58723R2

Fascia Iliaca Compartment Block for Surgical Analgesia in Children with Osteogenesis Imperfecta: A Retrospective Cohort Study

Dear editor Robert Jeenchen Chen,

We hope this message finds you well!

We sincerely express our gratitude for your review and guidance on our manuscript, as well as those of the three reviewers. We fully acknowledge that your professional knowledge can substantially improve the quality of the manuscript. We are aware that these are aspects that can be revised, and we are of the view that with certain revisions, our manuscript can attain a high - quality standard appropriate for publication.

Below is our point-by-point response to the comments, along with the corresponding revisions. The modified content in the manuscript will be highlighted in red.

Please feel free to contact us if you have any questions during the revision process.

Best regards,

Xueli Jiang

Journal Requirements:

If the reviewer comments include a recommendation to cite specific previously published works, please review and evaluate these publications to determine whether they are relevant and should be cited. There is no requirement to cite these works unless the editor has indicated otherwise.

Response: We extend our sincere gratitude to the editors for providing guidelines on handling reviewers' citation requirements. Throughout the manuscript revision process, we strictly adhered to the relevant provisions of these guidelines. During this review cycle, no citation-related modification suggestions were received from the reviewers, thus no adjustments were necessary. We will continue to uphold these guidelines and implement them rigorously in our future research endeavors.

Revision: None.

2. Please review your reference list to ensure that it is complete and correct. If you have cited papers that have been retracted, please include the rationale for doing so in the manuscript text, or remove these references and replace them with relevant current references. Any changes to the reference list should be mentioned in the rebuttal letter that accompanies your revised manuscript. If you need to cite a retracted article, indicate the article's retracted status in the References list and also include a citation and full reference for the retraction notice.

Response: We extend our sincere gratitude to the editor for reminding us of the integrity and accuracy of the reference formatting. We have carried out a thorough review of all references in the manuscript, meticulously verifying the accuracy of each entry, which encompasses author names, titles, publication journals, volume/issue/page numbers, and publication years. **All cited references are confirmed to be valid, formally published studies that have not been retracted, and no references have been cited from retracted publications.** In case such issues emerge in the future, we will strictly comply with the required labeling and explanations. During this review, we added the DOI and PMID for certain references to guarantee format consistency, and all corrections are highlighted in red in the revised manuscript.

Revision: Some references include DOI and PMID.

Reviewers' comments:

Reviewer #1: All comments have been addressed.

The manuscript describes a technically sound piece of scientific research with data that supports the conclusions.

Response: We sincerely appreciate the positive affirmation of our manuscript from you. We are very glad that all our previous revisions have been recognized and accepted.

Revision: None.

Reviewer #2: Thank you for replying to the observations. However, it is still not convincing considering the study design. Comparison in retrospective design with significant limitations failed to convey the message.

Response: We sincerely appreciate your identification of this critical issue. We fully recognize that the retrospective cohort design employed in this study inherently possesses limitations compared to prospective randomized controlled trials, which may partially compromise the reliability of the evidence. To address this concern, we have repeatedly elucidated the design limitations in the discussion section of the paper and explicitly stated that the study's conclusions require further validation through large-scale prospective studies in the future.

Meanwhile, we have elaborated in detail on the rationale for adopting a retrospective design: given that children with osteogenesis imperfecta constitute a rare population, large-scale prospective randomized controlled trials are difficult to conduct in the short term; whereas retrospective studies can utilize existing clinical data to provide preliminary clinical evidence for the application of iliac fascial compartment block in this population, thereby laying the foundation for subsequent high-quality research. We sincerely apologize for the insufficient elaboration of this aspect in previous versions and hope that this supplementary explanation will enhance the rigor and persuasiveness of our argument.

Revision: The Discussion section has been enhanced to elaborate on the rationale and limitations of this retrospective study design, as well as its clinical value.

Reviewer #3: Thank you for making appropriate changes. As long as the discussion falls into word limits I have no further changes.

Response: We sincerely thank you for your approval of our revisions and for your valuable reminder regarding the word limit for the discussion section. We have carefully reviewed the word count in the revised discussion section and ensured it strictly complies with the specified limits.

Revision: None.
