# Supplementary material for: Fascia iliaca compartment block for surgical analgesia in children with osteogenesis imperfecta: A retrospective cohort study
Source: PLoS One. 2026 Jul 2;21(7):e0353186. doi: 10.1371/journal.pone.0353186 (PMC13327246; doi:10.1371/journal.pone.0353186)
Supplement: Attachment — Submitted filename: Response to Reviewers 0520.pdf [file pone.0353186.s005.pdf]

PONE-D-25-58723R3

Fascia Iliaca Compartment Block for Surgical Analgesia in Children with Osteogenesis Imperfecta: A Retrospective Cohort Study

Dear Mr Robert Jeenchen Chen,

We hope this message finds you well!

We sincerely express our gratitude for your review and guidance on our manuscript, as well as those of all reviewers.

The research team has conducted multiple revisions to this manuscript, addressing the majority of identified issues. However, consensus regarding research bias remains elusive with Professor Jamshed Akhtar. Our team highly commends his rigorous academic approach; some of his recommendations have been adopted, while those not adopted are explained in detail. Furthermore, the manuscript has been revised again to further minimize research bias. We hope these improvements will be recognized.

Below is our point-by-point response to the comments, along with the corresponding revisions. The modified content in the manuscript will be highlighted in red.

Please feel free to contact us if you have any questions during the revision process.

Best regards,

Xueli Jiang

Reviewer #1: All comments have been addressed. The manuscript describes a technically sound piece of scientific research with data that supports the conclusions. The conclusions are based on the data presented.

Response: We sincerely appreciate the reviewer's positive comments and recognition of our work. We would like to express our sincere gratitude for the reviewer's valuable guidance and affirmation that helped us improve the quality of this manuscript.

Revision: No revision is required for this comment.

Reviewer #2: In present form the submission still lacks vigor. The data although supported by number and figures, however, bias is still apparent. This may be addressed by removing comparison arm.

Response:

Dear Prof. Jamshed Akhtar,

Thank you for your insightful remarks regarding potential biases in our study design. We have recognized your consistent commitment to scientific rigor. We understand the concerns about potential biases introduced by comparative groups and recommended removing the control group; however, we consider this unacceptable. Firstly, the control group is essential for addressing our primary research questions. By providing necessary contextual information for interpreting our findings, the comparative group enhances rather than undermines the persuasiveness of our study. Although retrospective studies inherently carry risks of bias, we have implemented multiple measures to minimize such biases, including blinded assessment of outcomes, use of validated measurement tools, and pre-registration of the study protocol. Secondly, this study has undergone ethical review and registration; therefore, we cannot arbitrarily modify the primary study protocol but can only optimize the data analysis methodology.

To address your concerns regarding potential bias, we have incorporated additional methods such as propensity score matching in the revised manuscript to minimize bias. To maintain research consistency, we have retained the results prior to matching, and we hope for your approval of these changes. We have also reiterated the study's limitations in the discussion section to facilitate objective evaluation of the findings by readers. Once again, we appreciate your valuable feedback, which has significantly contributed to refining this research.

Revision: The method section has added content on propensity score matching. Added propensity score matching analysis results in the Results section, supplemented a detailed description of research limitations and bias control measures in the Discussion section, and all modifications are marked in red in the manuscript.
