# Supplementary material for: Fascia iliaca compartment block for surgical analgesia in children with osteogenesis imperfecta: A retrospective cohort study
Source: PLoS One. 2026 Jul 2;21(7):e0353186. doi: 10.1371/journal.pone.0353186 (PMC13327246; doi:10.1371/journal.pone.0353186)
Supplement: Peer review history

# Peer review: Decision Letter 0

**DOI:** 10.1371/journal.pone.0353186.r001

**Authors:** Robert Chen

19 Jan 2026

PONE-D-25-58723

Fascia Iliaca Compartment Block for Surgical Analgesia in Children with Osteogenesis Imperfecta: A Retrospective Cohort Study

PLOS One

Dear Dr. Jiang,

Thank you for submitting your manuscript to PLOS ONE. After careful consideration, we feel that it has merit but does not fully meet PLOS ONE’s publication criteria as it currently stands. Therefore, we invite you to submit a revised version of the manuscript that addresses the points raised during the review process.

Revise.

Please submit your revised manuscript by Mar 05 2026 11:59PM. If you will need more time than this to complete your revisions, please reply to this message or contact the journal office at plosone@plos.org. When you're ready to submit your revision, log on to https://www.editorialmanager.com/pone/ and select the 'Submissions Needing Revision' folder to locate your manuscript file.

Please include the following items when submitting your revised manuscript:

- A letter that responds to each point raised by the academic editor and reviewer(s). You should upload this letter as a separate file labeled 'Response to Reviewers'.
- A marked-up copy of your manuscript that highlights changes made to the original version. You should upload this as a separate file labeled 'Revised Manuscript with Track Changes'.
- An unmarked version of your revised paper without tracked changes. You should upload this as a separate file labeled 'Manuscript'.

If you would like to make changes to your financial disclosure, please include your updated statement in your cover letter. Guidelines for resubmitting your figure files are available below the reviewer comments at the end of this letter.

If applicable, we recommend that you deposit your laboratory protocols in protocols.io to enhance the reproducibility of your results. Protocols.io assigns your protocol its own identifier (DOI) so that it can be cited independently in the future. For instructions see: https://journals.plos.org/plosone/s/submission-guidelines#loc-laboratory-protocols. Additionally, PLOS ONE offers an option for publishing peer-reviewed Lab Protocol articles, which describe protocols hosted on protocols.io. Read more information on sharing protocols at https://plos.org/protocols?utm_medium=editorial-email&utm_source=authorletters&utm_campaign=protocols.

We look forward to receiving your revised manuscript.

Kind regards,

Robert Jeenchen Chen, MD, MPH, ChFC®, EA

Academic Editor

PLOS One

Journal Requirements:

When submitting your revision, we need you to address these additional requirements.

1. Please ensure that your manuscript meets PLOS ONE's style requirements, including those for file naming. The PLOS ONE style templates can be found at

https://journals.plos.org/plosone/s/file?id=wjVg/PLOSOne_formatting_sample_main_body.pdf and

https://journals.plos.org/plosone/s/file?id=ba62/PLOSOne_formatting_sample_title_authors_affiliations.pdf.

2. Please provide additional details regarding participant consent. In the ethics statement in the Methods and online submission information, please ensure that you have specified (1) whether consent was informed and (2) what type you obtained (for instance, written or verbal, and if verbal, how it was documented and witnessed). If your study included minors, state whether you obtained consent from parents or guardians. If the need for consent was waived by the ethics committee, please include this information.

If you are reporting a retrospective study of medical records or archived samples, please ensure that you have discussed whether all data were fully anonymized before you accessed them and/or whether the IRB or ethics committee waived the requirement for informed consent. If patients provided informed written consent to have data from their medical records used in research, please include this information.

3. Thank you for stating in your Funding Statement:

“This study was financially supported by the Natural Science Foundation of Jiangsu Province (BK20241800)”

Please provide an amended statement that declares *all* the funding or sources of support (whether external or internal to your organization) received during this study, as detailed online in our guide for authors at http://journals.plos.org/plosone/s/submit-now.  Please also include the statement “There was no additional external funding received for this study.” in your updated Funding Statement.

Please include your amended Funding Statement within your cover letter. We will change the online submission form on your behalf.

4. Thank you for stating the following financial disclosure:

“This study was financially supported by the Natural Science Foundation of Jiangsu Province (BK20241800)”

Please state what role the funders took in the study.  If the funders had no role, please state: "The funders had no role in study design, data collection and analysis, decision to publish, or preparation of the manuscript."

If this statement is not correct you must amend it as needed.

Please include this amended Role of Funder statement in your cover letter; we will change the online submission form on your behalf.

5. We note that your Data Availability Statement is currently as follows: [All relevant data are within the manuscript and its Supporting Information files.]

Please confirm at this time whether or not your submission contains all raw data required to replicate the results of your study. Authors must share the “minimal data set” for their submission. PLOS defines the minimal data set to consist of the data required to replicate all study findings reported in the article, as well as related metadata and methods (https://journals.plos.org/plosone/s/data-availability#loc-minimal-data-set-definition).

For example, authors should submit the following data:

- The values behind the means, standard deviations and other measures reported;

- The values used to build graphs;

- The points extracted from images for analysis.

Authors do not need to submit their entire data set if only a portion of the data was used in the reported study.

If our submission does not contain these data, please either upload them as Supporting Information files or deposit them to a stable, public repository and provide us with the relevant URLs, DOIs, or accession numbers. For a list of recommended repositories, please see https://journals.plos.org/plosone/s/recommended-repositories.

If there are ethical or legal restrictions on sharing a de-identified data set, please explain them in detail (e.g., data contain potentially sensitive information, data are owned by a third-party organization, etc.) and who has imposed them (e.g., an ethics committee). Please also provide contact information for a data access committee, ethics committee, or other institutional body to which data requests may be sent. If data are owned by a third party, please indicate how others may request data access.

6. If the reviewer comments include a recommendation to cite specific previously published works, please review and evaluate these publications to determine whether they are relevant and should be cited. There is no requirement to cite these works unless the editor has indicated otherwise.

[Note: HTML markup is below. Please do not edit.]

Reviewers' comments:

Reviewer's Responses to Questions

**Comments to the Author**

1. Is the manuscript technically sound, and do the data support the conclusions?

The manuscript must describe a technically sound piece of scientific research with data that supports the conclusions. Experiments must have been conducted rigorously, with appropriate controls, replication, and sample sizes. The conclusions must be drawn appropriately based on the data presented.

Reviewer #1: Yes

Reviewer #2: Partly

**********

2. Has the statistical analysis been performed appropriately and rigorously?

Reviewer #1: Yes

Reviewer #2: I Don't Know

**********

3. Have the authors made all data underlying the findings in their manuscript fully available?

The PLOS Data policy requires authors to make all data underlying the findings described in their manuscript fully available without restriction, with rare exception (please refer to the Data Availability Statement in the manuscript PDF file). The data should be provided as part of the manuscript or its supporting information, or deposited to a public repository. For example, in addition to summary statistics, the data points behind means, medians and variance measures should be available. If there are restrictions on publicly sharing data—e.g. participant privacy or use of data from a third party—those must be specified.

Reviewer #1: Yes

Reviewer #2: Yes

**********

4. Is the manuscript presented in an intelligible fashion and written in standard English?

PLOS ONE does not copyedit accepted manuscripts, so the language in submitted articles must be clear, correct, and unambiguous. Any typographical or grammatical errors should be corrected at revision, so please note any specific errors here.

Reviewer #1: Yes

Reviewer #2: Yes

**********

5. Review Comments to the Author

Please use the space provided to explain your answers to the questions above. You may also include additional comments for the author, including concerns about dual publication, research ethics, or publication ethics. (Please upload your review as an attachment if it exceeds 20,000 characters)

Reviewer #1: The manuscript addresses an important and underexplored topic - perioperative analgesia in children with osteogenesis imperfecta (OI), a rare condition associated with significant anesthetic and postoperative pain management challenges. The use of fascia iliaca compartment block (FICB) in this vulnerable population is clinically relevant and potentially impactful, given the need to minimize opioid exposure, reduce pain-related stress, and avoid complications related to fragile bones.

The study provides valuable preliminary data suggesting that FICB may be an effective and safe component of multimodal analgesia in pediatric patients with OI undergoing orthopedic surgery. The manuscript is generally well written, clearly structured, and easy to follow. However, several methodological, analytical, and reporting issues should be addressed to strengthen the scientific rigor and interpretability of the findings.

1. The manuscript would benefit from a clearer explanation of how patients were selected for receiving FICB versus standard analgesia.

2. It remains unclear whether the decision to perform FICB was influenced by patient characteristics, surgeon preference, anesthesiologist experience, or temporal changes in practice, which introduces a potential selection bias.

3. Confounding factors (e.g., fracture severity, surgical complexity, OI subtype, baseline pain sensitivity) are not sufficiently explored.

4. Please clarify patient selection or expand the limitations section to explicitly discuss selection bias, confounding by indication, and the absence of randomization.

5. OI encompasses a wide spectrum of severity (types I–V and beyond), which may significantly affect pain perception, fracture burden, and postoperative recovery. It is unclear whether disease severity or subtype was comparable between groups. If available, provide data on OI type, clinical severity, or surrogate markers (e.g., fracture history, bisphosphonate therapy). If not available, this should be explicitly stated as a limitation.

6. The timing of the block (preoperative vs postoperative), local anesthetic concentration and volume, and use of ultrasound guidance should be described in greater detail.It is unclear whether intraoperative and postoperative systemic analgesic regimens were standardized between groups.

Provide a clearer description of the analgesic protocol and clarify whether other analgesics (e.g., acetaminophen, NSAIDs, opioids) were administered in a standardized manner.

7. Pain scores and opioid consumption are appropriate primary outcomes; however, their assessment raises several concerns. The manuscript should clarify whether validated, age-appropriate pain scales were consistently used across all patients.

8. The timing of pain assessments (e.g., PACU, first 24 hours, later postoperative period) should be clearly specified. Opioid consumption should ideally be normalized (e.g., morphine equivalents per kg) to allow meaningful comparison.

Clarify pain assessment methodology and provide justification for the chosen outcome time points.

Reviewer #2: The study is interesting. However, as authors themselves mentioned huge limitations of the results, the importance and significance are significantly compromised. The very nature of being retrospective and spanned over nearly six years with small number of patients, unequal groups and significant selection bias raise red flags. Why cohort study design is mentioned needs clarity. It is also strange that mean values of age and BMI are almost similar. It can not be a coincidence.

Pediatric anesthesia is safe and multimodal pain therapy is a routine process. In this study ultrasound guided block is added and it is expected to improve pain. There is no new information in that context.

It is also come as a surprise that over six years all patients were subjected to large number investigation / tests like CRP and others which are not performed routinely. What may be the reason for this?

Reference citation is also not appropriate. Large number of references are not required.

Reference number 8 cited in methods section is of modified radical mastectomy and has no relevance. There is verbosity and unnecessary comparison of variables while objective could have been addressed by clinical observations done objectively.

**********

6. PLOS authors have the option to publish the peer review history of their article (what does this mean?). If published, this will include your full peer review and any attached files.

If you choose “no”, your identity will remain anonymous but your review may still be made public.

**Do you want your identity to be public for this peer review?**  For information about this choice, including consent withdrawal, please see our Privacy Policy.

Reviewer #1: **Yes:** Oksana Boyarchuk

Reviewer #2: **Yes:** JAMSHED AKHTAR

**********

[NOTE: If reviewer comments were submitted as an attachment file, they will be attached to this email and accessible via the submission site. Please log into your account, locate the manuscript record, and check for the action link "View Attachments". If this link does not appear, there are no attachment files.]

To ensure your figures meet our technical requirements, please review our figure guidelines: https://journals.plos.org/plosone/s/figures

You may also use PLOS’s free figure tool, NAAS, to help you prepare publication quality figures: https://journals.plos.org/plosone/s/figures#loc-tools-for-figure-preparation.

NAAS will assess whether your figures meet our technical requirements by comparing each figure against our figure specifications.

# Author comment: Author response to Decision Letter 1

**DOI:** 10.1371/journal.pone.0353186.r002

22 Feb 2026

PONE-D-25-58723

Fascia Iliaca Compartment Block for Surgical Analgesia in Children with Osteogenesis Imperfecta: A Retrospective Cohort Study

Dear editor Robert Jeenchen Chen,

We hope this message finds you well! And we wish you a happy Chinese Spring Festival!

We sincerely appreciate your review and guidance on our manuscript, as well as that of the two reviewers. We fully recognize that your expertise can significantly enhance the quality of the manuscript. We understand that these are revisable aspects, and we believe that with some revisions, our manuscript can reach a high-quality standard suitable for publication.

Below is our point-by-point response to the comments, along with corresponding revisions. Highlight the modified content in red in the manuscript.

Please do not hesitate to contact us if you encounter any questions during the revision process.

Best regards,

Xueli Jiang

Journal Requirements:

When submitting your revision, we need you to address these additional requirements.

1. Please ensure that your manuscript meets PLOS ONE's style requirements, including those for file naming. The PLOS ONE style templates can be found at

https://journals.plos.org/plosone/s/file?id=wjVg/PLOSOne_formatting_sample_main_body.pdf and

https://journals.plos.org/plosone/s/file?id=ba62/PLOSOne_formatting_sample_title_authors_affiliations.pdf.

Response: We have revised the article to ensure that it conforms to the journal format requirements.

2. Please provide additional details regarding participant consent. In the ethics statement in the Methods and online submission information, please ensure that you have specified (1) whether consent was informed and (2) what type you obtained (for instance, written or verbal, and if verbal, how it was documented and witnessed). If your study included minors, state whether you obtained consent from parents or guardians. If the need for consent was waived by the ethics committee, please include this information.

If you are reporting a retrospective study of medical records or archived samples, please ensure that you have discussed whether all data were fully anonymized before you accessed them and/or whether the IRB or ethics committee waived the requirement for informed consent. If patients provided informed written consent to have data from their medical records used in research, please include this information.

Response: This study is a retrospective study that has undergone ethical review and was exempt from informed consent.

Revision: Add a statement in the methods section that informed consent was waived (lines ...).

3. Thank you for stating in your Funding Statement:

"This study was financially supported by the Natural Science Foundation of Jiangsu Province (BK20241800)"

Please provide an amended statement that declares *all* the funding or sources of support (whether external or internal to your organization) received during this study, as detailed online in our guide for authors at http://journals.plos.org/plosone/s/submit-now.  Please also include the statement "There was no additional external funding received for this study." in your updated Funding Statement.

Please include your amended Funding Statement within your cover letter. We will change the online submission form on your behalf.

Response: We have supplemented the Funding Statement as required and included the statement in the cover letter.

4. Thank you for stating the following financial disclosure:

"This study was financially supported by the Natural Science Foundation of Jiangsu Province (BK20241800)"

Please state what role the funders took in the study.  If the funders had no role, please state: "The funders had no role in study design, data collection and analysis, decision to publish, or preparation of the manuscript."

If this statement is not correct you must amend it as needed.

Please include this amended Role of Funder statement in your cover letter; we will change the online submission form on your behalf.

Response: We have supplemented the Funding Statement as required and included the statement in the cover letter.

5. We note that your Data Availability Statement is currently as follows: [All relevant data are within the manuscript and its Supporting Information files.]

Please confirm at this time whether or not your submission contains all raw data required to replicate the results of your study. Authors must share the "minimal data set" for their submission. PLOS defines the minimal data set to consist of the data required to replicate all study findings reported in the article, as well as related metadata and methods (https://journals.plos.org/plosone/s/data-availability#loc-minimal-data-set-definition).

For example, authors should submit the following data:

- The values behind the means, standard deviations and other measures reported;

- The values used to build graphs;

- The points extracted from images for analysis.

Authors do not need to submit their entire data set if only a portion of the data was used in the reported study.

If our submission does not contain these data, please either upload them as Supporting Information files or deposit them to a stable, public repository and provide us with the relevant URLs, DOIs, or accession numbers. For a list of recommended repositories, please see https://journals.plos.org/plosone/s/recommended-repositories.

If there are ethical or legal restrictions on sharing a de-identified data set, please explain them in detail (e.g., data contain potentially sensitive information, data are owned by a third-party organization, etc.) and who has imposed them (e.g., an ethics committee). Please also provide contact information for a data access committee, ethics committee, or other institutional body to which data requests may be sent. If data are owned by a third party, please indicate how others may request data access.

Response: All statistical results are included in our manuscript. Additionally, our raw data are uploaded as Supporting Information files during the revision process.

To Reviewer #1,

We hope this message finds you well! And we wish you a happy Chinese Spring Festival!

We sincerely appreciate your review and guidance on our manuscript. We fully recognize that your expertise can significantly enhance the quality of the manuscript. We understand that these are revisable aspects, and we believe that with some revisions, our manuscript can reach a high-quality standard suitable for publication.

Below is our point-by-point response to the comments, along with corresponding revisions. We have put together some of the relevant comments to respond to.

Please do not hesitate to contact us if you encounter any questions during the revision process.

Best regards,

Corresponding author (XJ)

1. The manuscript would benefit from a clearer explanation of how patients were selected for receiving FICB versus standard analgesia.

2. It remains unclear whether the decision to perform FICB was influenced by patient characteristics, surgeon preference, anesthesiologist experience, or temporal changes in practice, which introduces a potential selection bias.

Response: Thank you for your review and guidance. This study is a retrospective study designed to compare the efficacy of FICB in the management of OI patients undergoing surgery. Given the rarity of OI surgical cases in clinical practice and the extended observation period, it was challenging to design a fully randomized prospective study. FCIB is a well-established clinical technique. Prior to surgery, anesthesiologists (rather than investigators) determine whether a patient is suitable for FICB analgesia based on comprehensive factors such as the patient's specific condition, and thoroughly communicate with the patient and their family. The final choice of analgesic method is entirely determined by the patient and their family. All clinical data for the patients in this study were retrospectively collected from medical records, and the anesthesiologists' decision-making process was based on clinical experience and the patient's actual condition, without any intervention by the investigators.

Regarding potential selection bias, although anesthesiologists' decisions may be influenced by multiple factors, we implemented a series of measures during the data analysis phase to minimize the impact of such bias. We compared patients' baseline characteristics and surgical-related information to ensure comparability between groups, thereby enabling an accurate assessment of the efficacy difference between FICB and standard analgesia in OI patients undergoing surgery. Additionally, we acknowledge the inherent limitations of retrospective studies, which cannot completely eliminate all potential selection biases. However, in the context of research on rare diseases, retrospective studies can provide valuable clinical evidence. In the future, we plan to conduct prospective randomized controlled trials to further clarify the optimal application and efficacy of FICB in OI patients undergoing surgery, employing more rigorous study designs to avoid potential bias issues.

Revision: Add a detailed description of 'Patient Selection/Grouping Criteria' in the 'Methods' section. Expand the content in the 'Discussion/Limitations' section to candidly discuss the inherent selection bias (Selection Bias) and indication confounding (Confounding by Indication) issues in retrospective studies.

3. Confounding factors (e.g., fracture severity, surgical complexity, OI subtype, baseline pain sensitivity) are not sufficiently explored.

4.Please clarify patient selection or expand the limitations section to explicitly discuss selection bias, confounding by indication, and the absence of randomization.

5. OI encompasses a wide spectrum of severity (types I–V and beyond), which may significantly affect pain perception, fracture burden, and postoperative recovery. It is unclear whether disease severity or subtype was comparable between groups. If available, provide data on OI type, clinical severity, or surrogate markers (e.g., fracture history, bisphosphonate therapy). If not available, this should be explicitly stated as a limitation.

Response: Thank you for your review and guidance. We recognize that confounding factors play a crucial role in the study's results. Due to the nature of this retrospective study and the limited data sources from medical records, comprehensive exploration of all confounding factors was a challenge. Nevertheless, we can confirm that we did make efforts to collect and analyze information on these factors as much as possible. The OI type was relatively easy to obtain. We have supplemented it into the baseline information, performed inter-group comparisons, and verified the inter-group balance.

However, due to insufficiently standardized and detailed documentation in medical records regarding fracture severity and surgical complexity, we have difficulty obtaining comprehensive and accurate data. This indeed represents a significant limitation in the current study. In subsequent research, we will focus on improving data collection methods and standards to ensure more thorough acquisition of this critical information.

Regarding baseline pain sensitivity, this is a challenging metric to precisely quantify, and there is a lack of objective documentation in medical records. We recognize that this factor may influence the study outcomes, but due to the retrospective nature of this study and the limitations of available data, we were unable to thoroughly investigate it. In future prospective studies, we will employ appropriate assessment tools and methods to accurately evaluate and analyze patients' baseline pain sensitivity.

Regarding the comparability of disease severity or subtypes between the two groups, although we have supplemented the information on OI types and conducted intergroup balance validation, we are also aware that this may not fully represent the severity of the disease. If there are additional data on clinical severity or surrogate markers, such as fracture history or bisphosphonate therapy, we will further collect and analyze them, and supplement the results in the study. If such data cannot be obtained, we will explicitly state this as a limitation of the study in the manuscript to ensure the scientific rigor and integrity of the research. Simultaneously, we will strive to improve the data collection system in future studies to minimize limitations caused by data gaps, thereby providing more reliable clinical evidence for the application of FICB in OI patients during surgery.

Revision: The comparison of OI typing was added in the results section and Table 1. In the limitations section, the absence of certain key baseline data and its implications were stated.

6. The timing of the block (preoperative vs postoperative), local anesthetic concentration and volume, and use of ultrasound guidance should be described in greater detail. It is unclear whether intraoperative and postoperative systemic analgesic regimens were standardized between groups.

Provide a clearer description of the analgesic protocol and clarify whether other analgesics (e.g., acetaminophen, NSAIDs, opioids) were administered in a standardized manner.

Response: We sincerely appreciate your valuable suggestions. The detailed procedure of ultrasound-guided FICB, including the block time point, local anesthetic concentration, and dosage, is provided in the Methods section.

As for the standardization of intraoperative and postoperative systemic analgesic regimens between groups, we understand that the lack of clarity was a concern. After a thorough review of the medical records, we found that while there was a general protocol in place, there were some variations in the actual administration of analgesics due to individual patient needs. We will clearly describe the general analgesic protocol in the manuscript and also mention the possible variations that occurred.

We will also clarify the use of other analgesics, such as acetaminophen. We have data on the frequency and dosage of these medications administered to each patient, and we will present this information in a tabular form. If the administration of these analgesics was not fully standardized, we will state this as a limitation in the study and discuss its potential impact on the results. To this end, we have added a chapter to the Method section to elaborate on this. However, due to the inability to document oral acetaminophen use in detail, the evaluation of postoperative efficacy was not considered a primary outcome in this study.

In future studies, we will ensure that all these details are carefully recorded and reported from the start to avoid any ambiguity and to provide more reliable evidence for clinical practice. We believe that these revisions will enhance the quality and clarity of our manuscript and address your concerns effectively.

Revision: The Methods section was expanded to include the implementation of postoperative analgesia. The Limitations section included a statement regarding the potential impact of missing data on postoperative analgesia (oral analgesics) on the evaluation results.

7. Pain scores and opioid consumption are appropriate primary outcomes; however, their assessment raises several concerns. The manuscript should clarify whether validated, age-appropriate pain scales were consistently used across all patients.

8. The timing of pain assessments (e.g., PACU, first 24 hours, later postoperative period) should be clearly specified. Opioid consumption should ideally be normalized (e.g., morphine equivalents per kg) to allow meaningful comparison.

Clarify pain assessment methodology and provide justification for the chosen outcome time points.

Response: We acknowledge the importance of these concerns. The Visual Analogue Scale (VAS) was feasible and widely used in clinical practice among the 6~15 year-old population enrolled in this study. As a retrospective study, the collected clinical data were assessed using VAS scores, which could not be altered. The manuscript provided the time points for evaluation. Finally, to more effectively compare differences in analgesic efficacy, the study results included not only the to

Attachment Submitted filename: Response to Reviewers.pdf

# Peer review: Decision Letter 1

**DOI:** 10.1371/journal.pone.0353186.r003

**Authors:** Robert Chen, Robert Chen

17 Mar 2026

PONE-D-25-58723R1

Fascia Iliaca Compartment Block for Surgical Analgesia in Children with Osteogenesis Imperfecta: A Retrospective Cohort Study

PLOS One

Dear Dr. Jiang,

Thank you for submitting your manuscript to PLOS ONE. After careful consideration, we feel that it has merit but does not fully meet PLOS ONE’s publication criteria as it currently stands. Therefore, we invite you to submit a revised version of the manuscript that addresses the points raised during the review process.

Please submit your revised manuscript by May 01 2026 11:59PM. If you will need more time than this to complete your revisions, please reply to this message or contact the journal office at plosone@plos.org. When you're ready to submit your revision, log on to https://www.editorialmanager.com/pone/ and select the 'Submissions Needing Revision' folder to locate your manuscript file.

Please include the following items when submitting your revised manuscript:

- A letter that responds to each point raised by the academic editor and reviewer(s). You should upload this letter as a separate file labeled 'Response to Reviewers'.
- A marked-up copy of your manuscript that highlights changes made to the original version. You should upload this as a separate file labeled 'Revised Manuscript with Track Changes'.
- An unmarked version of your revised paper without tracked changes. You should upload this as a separate file labeled 'Manuscript'.

If you would like to make changes to your financial disclosure, please include your updated statement in your cover letter. Guidelines for resubmitting your figure files are available below the reviewer comments at the end of this letter.

If applicable, we recommend that you deposit your laboratory protocols in protocols.io to enhance the reproducibility of your results. Protocols.io assigns your protocol its own identifier (DOI) so that it can be cited independently in the future. For instructions see: https://journals.plos.org/plosone/s/submission-guidelines#loc-laboratory-protocols. Additionally, PLOS ONE offers an option for publishing peer-reviewed Lab Protocol articles, which describe protocols hosted on protocols.io. Read more information on sharing protocols at https://plos.org/protocols?utm_medium=editorial-email&utm_source=authorletters&utm_campaign=protocols.

We look forward to receiving your revised manuscript.

Kind regards,

Robert Jeenchen Chen, MD, MPH, ChFC®, EA

Academic Editor

PLOS One

Journal Requirements:

If the reviewer comments include a recommendation to cite specific previously published works, please review and evaluate these publications to determine whether they are relevant and should be cited. There is no requirement to cite these works unless the editor has indicated otherwise.

[Note: HTML markup is below. Please do not edit.]

Reviewers' comments:

Reviewer's Responses to Questions

**Comments to the Author**

1. If the authors have adequately addressed your comments raised in a previous round of review and you feel that this manuscript is now acceptable for publication, you may indicate that here to bypass the “Comments to the Author” section, enter your conflict of interest statement in the “Confidential to Editor” section, and submit your "Accept" recommendation.

Reviewer #1: All comments have been addressed

Reviewer #2: (No Response)

Reviewer #3: All comments have been addressed

**********

2. Is the manuscript technically sound, and do the data support the conclusions?

The manuscript must describe a technically sound piece of scientific research with data that supports the conclusions. Experiments must have been conducted rigorously, with appropriate controls, replication, and sample sizes. The conclusions must be drawn appropriately based on the data presented.

Reviewer #1: Yes

Reviewer #2: No

Reviewer #3: No

**********

3. Has the statistical analysis been performed appropriately and rigorously?

Reviewer #1: Yes

Reviewer #2: I Don't Know

Reviewer #3: Yes

**********

4. Have the authors made all data underlying the findings in their manuscript fully available?

The PLOS Data policy requires authors to make all data underlying the findings described in their manuscript fully available without restriction, with rare exception (please refer to the Data Availability Statement in the manuscript PDF file). The data should be provided as part of the manuscript or its supporting information, or deposited to a public repository. For example, in addition to summary statistics, the data points behind means, medians and variance measures should be available. If there are restrictions on publicly sharing data—e.g. participant privacy or use of data from a third party—those must be specified.

Reviewer #1: Yes

Reviewer #2: Yes

Reviewer #3: No

**********

5. Is the manuscript presented in an intelligible fashion and written in standard English?

PLOS ONE does not copyedit accepted manuscripts, so the language in submitted articles must be clear, correct, and unambiguous. Any typographical or grammatical errors should be corrected at revision, so please note any specific errors here.

Reviewer #1: Yes

Reviewer #2: No

Reviewer #3: Yes

**********

6. Review Comments to the Author

Please use the space provided to explain your answers to the questions above. You may also include additional comments for the author, including concerns about dual publication, research ethics, or publication ethics. (Please upload your review as an attachment if it exceeds 20,000 characters)

Reviewer #1: All comments have been addressed. The manuscript is technically sound, and the data support the conclusions.

Reviewer #2: The response is based upon large number of limitations for almost all the observations. This nullifies the purpose of research. Readers may not get an impression that are reading a compromised study.

Reviewer #3: The authors present a revised manuscript on the use of regional anesthesia in osteogenesis imperfecta patients. This is an interesting collection of cases and the paper is well-written. While I did not review the original manuscript, it seems that the authors have addressed the comments well.

My major concern with the manuscript is that the data are presented as if the study is designed to determine whether the block is effective or not. The study is not randomized or blinded, and patient allocation and intraoperative anesthetic were not controlled. It would therefore be more appropriate to present the results as a case series or feasibility study. This could be accomplished primarily by tempering the discussion to avoid arguing that the results support the effectiveness of the block.

**********

7. PLOS authors have the option to publish the peer review history of their article (what does this mean?). If published, this will include your full peer review and any attached files.

If you choose “no”, your identity will remain anonymous but your review may still be made public.

**Do you want your identity to be public for this peer review?**  For information about this choice, including consent withdrawal, please see our Privacy Policy.

Reviewer #1: **Yes:** Oksana Boyarchuk

Reviewer #2: **Yes:** Jamshed Akhtar

Reviewer #3: No

**********

[NOTE: If reviewer comments were submitted as an attachment file, they will be attached to this email and accessible via the submission site. Please log into your account, locate the manuscript record, and check for the action link "View Attachments". If this link does not appear, there are no attachment files.]

To ensure your figures meet our technical requirements, please review our figure guidelines: https://journals.plos.org/plosone/s/figures

You may also use PLOS’s free figure tool, NAAS, to help you prepare publication quality figures: https://journals.plos.org/plosone/s/figures#loc-tools-for-figure-preparation.

NAAS will assess whether your figures meet our technical requirements by comparing each figure against our figure specifications.

# Author comment: Author response to Decision Letter 2

**DOI:** 10.1371/journal.pone.0353186.r004

24 Mar 2026

PONE-D-25-58723R1

Fascia Iliaca Compartment Block for Surgical Analgesia in Children with Osteogenesis Imperfecta: A Retrospective Cohort Study

Dear editor Robert Jeenchen Chen,

We hope this message finds you well!

We sincerely appreciate your review and guidance on our manuscript, as well as that of the three reviewers. We fully recognize that your expertise can significantly enhance the quality of the manuscript. We understand that these are revisable aspects, and we believe that with some revisions, our manuscript can reach a high-quality standard suitable for publication.

Below is our point-by-point response to the comments, along with corresponding revisions. Highlight the modified content in red in the manuscript.

Please do not hesitate to contact us if you encounter any questions during the revision process.

Best regards,

Xueli Jiang

To Reviewer #1:

All comments have been addressed. The manuscript is technically sound, and the data support the conclusions.

Response: We are deeply grateful to you for your careful review and positive evaluation of our manuscript. Your confirmation that all comments have been addressed and that the manuscript is technically sound with data supporting the conclusions is extremely encouraging. We have taken this feedback to heart and are committed to maintaining this standard in the final version of the manuscript. The thoroughness of your assessment has provided us with confidence that our work meets the rigorous criteria of the journal. We have made sure to incorporate all suggested improvements and have double-checked the technical aspects and data interpretation to ensure the highest level of accuracy and validity. We believe that these efforts have strengthened the manuscript significantly, and we are thankful for your valuable input which has contributed to its refinement.

Revision: None.

To Reviewer #2:

The response is based upon large number of limitations for almost all the observations. This nullifies the purpose of research. Readers may not get an impression that are reading a compromised study.

Response: We sincerely appreciate your pointing out this critical concern, which has prompted us to thoroughly reflect on the presentation of limitations in our manuscript. We acknowledge that the initial emphasis on limitations might have overshadowed the potential value of our research, inadvertently creating an impression of a "compromised study." This was never our intention, and we deeply regret any confusion it may have caused.

First, we would like to clarify that while we have been transparent about the limitations—an essential aspect of scientific rigor—we do not view our study as inherently compromised. Instead, we believe it addresses an important gap in the literature, particularly regarding the use of FICB for surgical analgesia in a highly vulnerable and understudied population: children with OI. OI children present unique challenges in pain management due to their skeletal fragility, potential for opioid sensitivity, and the need for multiple surgeries. There is a paucity of data specifically examining regional analgesic techniques in this patient group, making even observational data valuable for guiding clinical practice.

To address your concern, we have revised the manuscript to better balance the discussion of limitations with a clearer articulation of the study's strengths and contributions. Specifically, we have:

1. Strengthened the introduction and discussion of the research gap: We have expanded on the rationale for studying FICB in OI children, emphasizing the unmet clinical need and the potential benefits of regional analgesia in this population (e.g., reducing opioid exposure and associated side effects). This provides a stronger foundation for why the study was conducted and its relevance despite its retrospective nature.

2. Reorganized the limitations section: Instead of presenting limitations upfront in a manner that might have seemed overwhelming, we have integrated a more measured discussion of limitations within the broader context of the study's findings and their implications. We have clearly distinguished between inherent limitations of retrospective cohort studies (e.g., potential selection bias, lack of randomization) and the specific steps we took to mitigate these (e.g., detailed chart review, inclusion of a comparator group where possible, sensitivity analyses).

3. Highlighted the study's strengths: We have explicitly stated the strengths, such as the focus on a rare and clinically challenging population, the relatively large sample size for such a specific cohort, the detailed collection of perioperative analgesic data, and the demonstration of FICB's feasibility and effectiveness in terms of pain scores and opioid reduction in this context.

4. Tempered the language regarding limitations: We have revised the language to avoid overly negative framing. For example, instead of stating that a limitation "nullifies" a finding, we now discuss how it "should be considered when interpreting" the result, and we offer suggestions for future research to address these limitations.

Our goal is to present a balanced and honest account of our work, one that acknowledges its constraints while also clearly communicating the novel insights it provides. We believe these revisions will provide readers with a more accurate and nuanced understanding of the study, allowing them to appreciate its contributions to clinical knowledge in this specialized area. We hope that with these changes, the manuscript will no longer convey the impression of a "compromised study" but rather a valuable, hypothesis-generating investigation that addresses an important clinical question.

5.Fact-based expression style: During revisions, we should candidly acknowledge the insufficient evidentiary strength of research findings and strive to avoid excessive interpretation and affirmative statements. Indeed, we fully concur with the importance of presenting the evidence strength of our research findings with the utmost honesty and avoiding over-interpretation or overly assertive claims. In light of this, we have meticulously revised the discussion section to ensure that our conclusions are firmly grounded in the observed data and that we refrain from making unwarranted extrapolations. Our aim is to present the data in a manner that is both transparent and responsible, allowing readers to form their own informed judgments about the implications of our work while acknowledging its inherent boundaries. This approach, we believe, is essential to maintaining scientific integrity and ensuring that our research contributes meaningfully to the existing body of knowledge without overstating its current scope or certainty.

Revision: We have made extensive revisions to the Discussion section, particularly the subsections on "Limitations" and the concluding paragraphs, to better balance the presentation of limitations with the study's strengths and clinical relevance. These revisions are highlighted in red in the manuscript.

Reviewer #3: The authors present a revised manuscript on the use of regional anesthesia in osteogenesis imperfecta patients. This is an interesting collection of cases and the paper is well-written. While I did not review the original manuscript, it seems that the authors have addressed the comments well.

My major concern with the manuscript is that the data are presented as if the study is designed to determine whether the block is effective or not. The study is not randomized or blinded, and patient allocation and intraoperative anesthetic were not controlled. It would therefore be more appropriate to present the results as a case series or feasibility study. This could be accomplished primarily by tempering the discussion to avoid arguing that the results support the effectiveness of the block.

Response: We sincerely appreciate your insightful observation and whole-heartedly concur with this crucial point. The retrospective, non-randomized, and non-blinded characteristics of our study, along with the lack of control over patient allocation and intraoperative anesthetics, truly prevent us from making conclusive claims about the efficacy of FICB in children with OI.

We recognize that our study was not designed to formally assess the effectiveness of the block as a randomized controlled trial would. However, while acknowledging the inherent limitations of these retrospective studies, we still recognize their significant value, albeit limited in scope.

In view of this, we have made substantial revisions to reframe the manuscript. Our primary focus is on depicting our experience with FICB in this specific patient population and exploring its potential role as part of a multimodal analgesic approach. Specifically, we have refined the language throughout the manuscript, especially in the Abstract, Results, and Discussion sections, to avoid suggesting causal efficacy. Instead of stating that "FICB is effective," we now stress that "our observations indicate that FICB might be a feasible and potentially advantageous component of perioperative pain management in OI children, meriting further investigation." We have eliminated any overly assertive claims regarding the block's effectiveness and instead spotlight the descriptive nature of our findings, the trends noted (such as decreased opioid consumption and improved pain scores in the FICB group), and their potential implications for clinical practice and future research design. Our current objective is to present these cases as valuable clinical experience that enriches the limited body of knowledge in this area and serves as a basis for generating hypotheses and devising more rigorous prospective studies to formally evaluate the efficacy and safety of FICB in OI children. These revisions are marked in red in the manuscript.

Revision: We have comprehensively reframed the manuscript to explicitly position it as a case series/feasibility study. This includes modifying revising the Abstract to clearly state the study's purpose as describing our experience with FICB in OI children and exploring its feasibility and potential role in multimodal analgesia, and adjusting the Introduction to emphasize the paucity of data in this area and the need for such descriptive reports. Throughout the Results and Discussion section, we have rephrased statements to focus on observed trends and descriptive statistics rather than inferential claims about effectiveness. The Discussion now centers on interpreting these observations within the context of the study's limitations, highlighting the feasibility and potential benefits suggested by the cases, and emphasizing the need for future controlled trials to confirm these preliminary findings. These extensive revisions are highlighted in red in the manuscript.

Attachment Submitted filename: Response to Reviewers 0323.pdf

# Peer review: Decision Letter 2

**DOI:** 10.1371/journal.pone.0353186.r005

**Authors:** Robert Chen, Robert Chen, Robert Chen

9 Apr 2026

PONE-D-25-58723R2

Fascia Iliaca Compartment Block for Surgical Analgesia in Children with Osteogenesis Imperfecta: A Retrospective Cohort Study

PLOS One

Dear Dr. Jiang,

Thank you for submitting your manuscript to PLOS ONE. After careful consideration, we feel that it has merit but does not fully meet PLOS ONE’s publication criteria as it currently stands. Therefore, we invite you to submit a revised version of the manuscript that addresses the points raised during the review process.

Please submit your revised manuscript by May 24 2026 11:59PM. If you will need more time than this to complete your revisions, please reply to this message or contact the journal office at plosone@plos.org. When you're ready to submit your revision, log on to https://www.editorialmanager.com/pone/ and select the 'Submissions Needing Revision' folder to locate your manuscript file.

Please include the following items when submitting your revised manuscript:

- A letter that responds to each point raised by the academic editor and reviewer(s). You should upload this letter as a separate file labeled 'Response to Reviewers'.
- A marked-up copy of your manuscript that highlights changes made to the original version. You should upload this as a separate file labeled 'Revised Manuscript with Track Changes'.
- An unmarked version of your revised paper without tracked changes. You should upload this as a separate file labeled 'Manuscript'.

If you would like to make changes to your financial disclosure, please include your updated statement in your cover letter. Guidelines for resubmitting your figure files are available below the reviewer comments at the end of this letter.

If applicable, we recommend that you deposit your laboratory protocols in protocols.io to enhance the reproducibility of your results. Protocols.io assigns your protocol its own identifier (DOI) so that it can be cited independently in the future. For instructions see: https://journals.plos.org/plosone/s/submission-guidelines#loc-laboratory-protocols. Additionally, PLOS ONE offers an option for publishing peer-reviewed Lab Protocol articles, which describe protocols hosted on protocols.io. Read more information on sharing protocols at https://plos.org/protocols?utm_medium=editorial-email&utm_source=authorletters&utm_campaign=protocols.

As the corresponding author, your ORCID iD is verified in the submission system and will appear in the published article. PLOS supports the use of ORCID, and we encourage all coauthors to register for an ORCID iD and use it as well. Please encourage your coauthors to verify their ORCID iD within the submission system before final acceptance, as unverified ORCID iDs will not appear in the published article. *Only*  the individual author can complete the verification step; PLOS staff *cannot*  verify ORCID iDs on behalf of authors.

We look forward to receiving your revised manuscript.

Kind regards,

Robert Jeenchen Chen, MD, MPH, ChFC®, EA

Academic Editor

PLOS One

**Journal Requirements:**

1. If the reviewer comments include a recommendation to cite specific previously published works, please review and evaluate these publications to determine whether they are relevant and should be cited. There is no requirement to cite these works unless the editor has indicated otherwise.

2. Please review your reference list to ensure that it is complete and correct. If you have cited papers that have been retracted, please include the rationale for doing so in the manuscript text, or remove these references and replace them with relevant current references. Any changes to the reference list should be mentioned in the rebuttal letter that accompanies your revised manuscript. If you need to cite a retracted article, indicate the article’s retracted status in the References list and also include a citation and full reference for the retraction notice.

**Additional Editor Comments:**

Revise.

[Note: HTML markup is below. Please do not edit.]

Reviewers' comments:

Reviewer's Responses to Questions

**Comments to the Author**

1. If the authors have adequately addressed your comments raised in a previous round of review and you feel that this manuscript is now acceptable for publication, you may indicate that here to bypass the “Comments to the Author” section, enter your conflict of interest statement in the “Confidential to Editor” section, and submit your "Accept" recommendation.

Reviewer #1: All comments have been addressed

Reviewer #2: (No Response)

Reviewer #3: All comments have been addressed

**********

2. Is the manuscript technically sound, and do the data support the conclusions?

The manuscript must describe a technically sound piece of scientific research with data that supports the conclusions. Experiments must have been conducted rigorously, with appropriate controls, replication, and sample sizes. The conclusions must be drawn appropriately based on the data presented.

Reviewer #1: Yes

Reviewer #2: No

Reviewer #3: Partly

**********

3. Has the statistical analysis been performed appropriately and rigorously?

Reviewer #1: Yes

Reviewer #2: I Don't Know

Reviewer #3: Yes

**********

4. Have the authors made all data underlying the findings in their manuscript fully available?

The PLOS Data policy requires authors to make all data underlying the findings described in their manuscript fully available without restriction, with rare exception (please refer to the Data Availability Statement in the manuscript PDF file). The data should be provided as part of the manuscript or its supporting information, or deposited to a public repository. For example, in addition to summary statistics, the data points behind means, medians and variance measures should be available. If there are restrictions on publicly sharing data—e.g. participant privacy or use of data from a third party—those must be specified.

Reviewer #1: Yes

Reviewer #2: Yes

Reviewer #3: Yes

**********

5. Is the manuscript presented in an intelligible fashion and written in standard English?

PLOS ONE does not copyedit accepted manuscripts, so the language in submitted articles must be clear, correct, and unambiguous. Any typographical or grammatical errors should be corrected at revision, so please note any specific errors here.

Reviewer #1: Yes

Reviewer #2: No

Reviewer #3: Yes

**********

6. Review Comments to the Author

Please use the space provided to explain your answers to the questions above. You may also include additional comments for the author, including concerns about dual publication, research ethics, or publication ethics. (Please upload your review as an attachment if it exceeds 20,000 characters)

**Reviewer #1:** All comments have been addressed.

The manuscript describes a technically sound piece of scientific research with data that supports the conclusions.

**Reviewer #2:** Thank you for replying to the observations. However, it is still not convincing considering the study design. Comparison in retrospective design with significant limitations failed to convey the message.

**Reviewer #3:**  Thank you for making appropriate changes. As long as the discussion falls into word limits I have no further changes.

**********

7. PLOS authors have the option to publish the peer review history of their article (what does this mean?). If published, this will include your full peer review and any attached files.

If you choose “no”, your identity will remain anonymous but your review may still be made public.

**Do you want your identity to be public for this peer review?**  For information about this choice, including consent withdrawal, please see our Privacy Policy.

Reviewer #1: No

Reviewer #2: No

Reviewer #3: No

**********

[NOTE: If reviewer comments were submitted as an attachment file, they will be attached to this email and accessible via the submission site. Please log into your account, locate the manuscript record, and check for the action link "View Attachments". If this link does not appear, there are no attachment files.]

To ensure your figures meet our technical requirements, please review our figure guidelines: https://journals.plos.org/plosone/s/figures

You may also use PLOS’s free figure tool, NAAS, to help you prepare publication quality figures: https://journals.plos.org/plosone/s/figures#loc-tools-for-figure-preparation.

NAAS will assess whether your figures meet our technical requirements by comparing each figure against our figure specifications.

# Author comment: Author response to Decision Letter 3

**DOI:** 10.1371/journal.pone.0353186.r006

4 May 2026

PONE-D-25-58723R2

Fascia Iliaca Compartment Block for Surgical Analgesia in Children with Osteogenesis Imperfecta: A Retrospective Cohort Study

Dear editor Robert Jeenchen Chen,

We hope this message finds you well!

We sincerely express our gratitude for your review and guidance on our manuscript, as well as those of the three reviewers. We fully acknowledge that your professional knowledge can substantially improve the quality of the manuscript. We are aware that these are aspects that can be revised, and we are of the view that with certain revisions, our manuscript can attain a high - quality standard appropriate for publication.

Below is our point-by-point response to the comments, along with the corresponding revisions. The modified content in the manuscript will be highlighted in red.

Please feel free to contact us if you have any questions during the revision process.

Best regards,

Xueli Jiang

Journal Requirements:

If the reviewer comments include a recommendation to cite specific previously published works, please review and evaluate these publications to determine whether they are relevant and should be cited. There is no requirement to cite these works unless the editor has indicated otherwise.

Response: We extend our sincere gratitude to the editors for providing guidelines on handling reviewers' citation requirements. Throughout the manuscript revision process, we strictly adhered to the relevant provisions of these guidelines. During this review cycle, no citation-related modification suggestions were received from the reviewers, thus no adjustments were necessary. We will continue to uphold these guidelines and implement them rigorously in our future research endeavors.

Revision: None.

2. Please review your reference list to ensure that it is complete and correct. If you have cited papers that have been retracted, please include the rationale for doing so in the manuscript text, or remove these references and replace them with relevant current references. Any changes to the reference list should be mentioned in the rebuttal letter that accompanies your revised manuscript. If you need to cite a retracted article, indicate the article’s retracted status in the References list and also include a citation and full reference for the retraction notice.

Response: We extend our sincere gratitude to the editor for reminding us of the integrity and accuracy of the reference formatting. We have carried out a thorough review of all references in the manuscript, meticulously verifying the accuracy of each entry, which encompasses author names, titles, publication journals, volume/issue/page numbers, and publication years. All cited references are confirmed to be valid, formally published studies that have not been retracted, and no references have been cited from retracted publications. In case such issues emerge in the future, we will strictly comply with the required labeling and explanations. During this review, we added the DOI and PMID for certain references to guarantee format consistency, and all corrections are highlighted in red in the revised manuscript.

Revision: Some references include DOI and PMID.

Reviewers' comments:

Reviewer #1: All comments have been addressed.

The manuscript describes a technically sound piece of scientific research with data that supports the conclusions.

Response: We sincerely appreciate the positive affirmation of our manuscript from you. We are very glad that all our previous revisions have been recognized and accepted.

Revision: None.

Reviewer #2: Thank you for replying to the observations. However, it is still not convincing considering the study design. Comparison in retrospective design with significant limitations failed to convey the message.

Response: We sincerely appreciate your identification of this critical issue. We fully recognize that the retrospective cohort design employed in this study inherently possesses limitations compared to prospective randomized controlled trials, which may partially compromise the reliability of the evidence. To address this concern, we have repeatedly elucidated the design limitations in the discussion section of the paper and explicitly stated that the study's conclusions require further validation through large-scale prospective studies in the future.

Meanwhile, we have elaborated in detail on the rationale for adopting a retrospective design: given that children with osteogenesis imperfecta constitute a rare population, large-scale prospective randomized controlled trials are difficult to conduct in the short term; whereas retrospective studies can utilize existing clinical data to provide preliminary clinical evidence for the application of iliac fascial compartment block in this population, thereby laying the foundation for subsequent high-quality research. We sincerely apologize for the insufficient elaboration of this aspect in previous versions and hope that this supplementary explanation will enhance the rigor and persuasiveness of our argument.

Revision: The Discussion section has been enhanced to elaborate on the rationale and limitations of this retrospective study design, as well as its clinical value.

Reviewer #3: Thank you for making appropriate changes. As long as the discussion falls into word limits I have no further changes.

Response: We sincerely thank you for your approval of our revisions and for your valuable reminder regarding the word limit for the discussion section. We have carefully reviewed the word count in the revised discussion section and ensured it strictly complies with the specified limits.

Revision: None.

Attachment Submitted filename: Response to Reviewers 0424.pdf

# Peer review: Decision Letter 3

**DOI:** 10.1371/journal.pone.0353186.r007

**Authors:** Robert Chen, Robert Chen, Robert Chen, Robert Chen

10 May 2026

PONE-D-25-58723R3

Fascia Iliaca Compartment Block for Surgical Analgesia in Children with Osteogenesis Imperfecta: A Retrospective Cohort Study

PLOS One

Dear Dr. Jiang,

Thank you for submitting your manuscript to PLOS ONE. After careful consideration, we feel that it has merit but does not fully meet PLOS ONE’s publication criteria as it currently stands. Therefore, we invite you to submit a revised version of the manuscript that addresses the points raised during the review process.

Revise.

Please submit your revised manuscript by Jun 24 2026 11:59PM. If you will need more time than this to complete your revisions, please reply to this message or contact the journal office at plosone@plos.org. When you're ready to submit your revision, log on to https://www.editorialmanager.com/pone/ and select the 'Submissions Needing Revision' folder to locate your manuscript file.

Please include the following items when submitting your revised manuscript:

- A letter that responds to each point raised by the academic editor and reviewer(s). You should upload this letter as a separate file labeled 'Response to Reviewers'.
- A marked-up copy of your manuscript that highlights changes made to the original version. You should upload this as a separate file labeled 'Revised Manuscript with Track Changes'.
- An unmarked version of your revised paper without tracked changes. You should upload this as a separate file labeled 'Manuscript'.

If you would like to make changes to your financial disclosure, please include your updated statement in your cover letter. Guidelines for resubmitting your figure files are available below the reviewer comments at the end of this letter.

If applicable, we recommend that you deposit your laboratory protocols in protocols.io to enhance the reproducibility of your results. Protocols.io assigns your protocol its own identifier (DOI) so that it can be cited independently in the future. For instructions see: https://journals.plos.org/plosone/s/submission-guidelines#loc-laboratory-protocols. Additionally, PLOS ONE offers an option for publishing peer-reviewed Lab Protocol articles, which describe protocols hosted on protocols.io. Read more information on sharing protocols at https://plos.org/protocols?utm_medium=editorial-email&utm_source=authorletters&utm_campaign=protocols.

As the corresponding author, your ORCID iD is verified in the submission system and will appear in the published article. PLOS supports the use of ORCID, and we encourage all coauthors to register for an ORCID iD and use it as well. Please encourage your coauthors to verify their ORCID iD within the submission system before final acceptance, as unverified ORCID iDs will not appear in the published article. *Only*  the individual author can complete the verification step; PLOS staff *cannot*  verify ORCID iDs on behalf of authors.

We look forward to receiving your revised manuscript.

Kind regards,

Robert Jeenchen Chen, MD, MPH, ChFC®, EA

Academic Editor

PLOS One

Journal Requirements:

If the reviewer comments include a recommendation to cite specific previously published works, please review and evaluate these publications to determine whether they are relevant and should be cited. There is no requirement to cite these works unless the editor has indicated otherwise.

[Note: HTML markup is below. Please do not edit.]

Reviewers' comments:

Reviewer's Responses to Questions

**Comments to the Author**

1. If the authors have adequately addressed your comments raised in a previous round of review and you feel that this manuscript is now acceptable for publication, you may indicate that here to bypass the “Comments to the Author” section, enter your conflict of interest statement in the “Confidential to Editor” section, and submit your "Accept" recommendation.

Reviewer #1: All comments have been addressed

Reviewer #2: (No Response)

**********

2. Is the manuscript technically sound, and do the data support the conclusions?

The manuscript must describe a technically sound piece of scientific research with data that supports the conclusions. Experiments must have been conducted rigorously, with appropriate controls, replication, and sample sizes. The conclusions must be drawn appropriately based on the data presented.

Reviewer #1: Yes

Reviewer #2: Partly

**********

3. Has the statistical analysis been performed appropriately and rigorously?

Reviewer #1: Yes

Reviewer #2: I Don't Know

**********

4. Have the authors made all data underlying the findings in their manuscript fully available?

The PLOS Data policy requires authors to make all data underlying the findings described in their manuscript fully available without restriction, with rare exception (please refer to the Data Availability Statement in the manuscript PDF file). The data should be provided as part of the manuscript or its supporting information, or deposited to a public repository. For example, in addition to summary statistics, the data points behind means, medians and variance measures should be available. If there are restrictions on publicly sharing data—e.g. participant privacy or use of data from a third party—those must be specified.

Reviewer #1: Yes

Reviewer #2: Yes

**********

5. Is the manuscript presented in an intelligible fashion and written in standard English?

PLOS ONE does not copyedit accepted manuscripts, so the language in submitted articles must be clear, correct, and unambiguous. Any typographical or grammatical errors should be corrected at revision, so please note any specific errors here.

Reviewer #1: Yes

Reviewer #2: Yes

**********

6. Review Comments to the Author

Please use the space provided to explain your answers to the questions above. You may also include additional comments for the author, including concerns about dual publication, research ethics, or publication ethics. (Please upload your review as an attachment if it exceeds 20,000 characters)

Reviewer #1: All comments have been addressed. The manuscript describes a technically sound piece of scientific research with data that supports the conclusions. The conclusions are based on the data presented.

Reviewer #2: In present form the submission still lacks vigor. The data although supported by number and figures, however, bias is still apparent. This may be addressed by removing comparison arm.

**********

7. PLOS authors have the option to publish the peer review history of their article (what does this mean?). If published, this will include your full peer review and any attached files.

If you choose “no”, your identity will remain anonymous but your review may still be made public.

**Do you want your identity to be public for this peer review?**  For information about this choice, including consent withdrawal, please see our Privacy Policy.

Reviewer #1: No

Reviewer #2: **Yes:** Jamshed Akhtar

**********

[NOTE: If reviewer comments were submitted as an attachment file, they will be attached to this email and accessible via the submission site. Please log into your account, locate the manuscript record, and check for the action link "View Attachments". If this link does not appear, there are no attachment files.]

To ensure your figures meet our technical requirements, please review our figure guidelines: https://journals.plos.org/plosone/s/figures

You may also use PLOS’s free figure tool, NAAS, to help you prepare publication quality figures: https://journals.plos.org/plosone/s/figures#loc-tools-for-figure-preparation.

NAAS will assess whether your figures meet our technical requirements by comparing each figure against our figure specifications.

# Author comment: Author response to Decision Letter 4

**DOI:** 10.1371/journal.pone.0353186.r008

22 May 2026

PONE-D-25-58723R3

Fascia Iliaca Compartment Block for Surgical Analgesia in Children with Osteogenesis Imperfecta: A Retrospective Cohort Study

Dear Mr Robert Jeenchen Chen,

We hope this message finds you well!

We sincerely express our gratitude for your review and guidance on our manuscript, as well as those of all reviewers.

The research team has conducted multiple revisions to this manuscript, addressing the majority of identified issues. However, consensus regarding research bias remains elusive with Professor Jamshed Akhtar. Our team highly commends his rigorous academic approach; some of his recommendations have been adopted, while those not adopted are explained in detail. Furthermore, the manuscript has been revised again to further minimize research bias. We hope these improvements will be recognized.

Below is our point-by-point response to the comments, along with the corresponding revisions. The modified content in the manuscript will be highlighted in red.

Please feel free to contact us if you have any questions during the revision process.

Best regards,

Xueli Jiang

Reviewer #1: All comments have been addressed. The manuscript describes a technically sound piece of scientific research with data that supports the conclusions. The conclusions are based on the data presented.

Response: We sincerely appreciate the reviewer's positive comments and recognition of our work. We would like to express our sincere gratitude for the reviewer's valuable guidance and affirmation that helped us improve the quality of this manuscript.

Revision: No revision is required for this comment.

Reviewer #2: In present form the submission still lacks vigor. The data although supported by number and figures, however, bias is still apparent. This may be addressed by removing comparison arm.

Response:

Dear Prof. Jamshed Akhtar,

Thank you for your insightful remarks regarding potential biases in our study design. We have recognized your consistent commitment to scientific rigor. We understand the concerns about potential biases introduced by comparative groups and recommended removing the control group; however, we consider this unacceptable. Firstly, the control group is essential for addressing our primary research questions. By providing necessary contextual information for interpreting our findings, the comparative group enhances rather than undermines the persuasiveness of our study. Although retrospective studies inherently carry risks of bias, we have implemented multiple measures to minimize such biases, including blinded assessment of outcomes, use of validated measurement tools, and pre-registration of the study protocol. Secondly, this study has undergone ethical review and registration; therefore, we cannot arbitrarily modify the primary study protocol but can only optimize the data analysis methodology.

To address your concerns regarding potential bias, we have incorporated additional methods such as propensity score matching in the revised manuscript to minimize bias. To maintain research consistency, we have retained the results prior to matching, and we hope for your approval of these changes. We have also reiterated the study's limitations in the discussion section to facilitate objective evaluation of the findings by readers. Once again, we appreciate your valuable feedback, which has significantly contributed to refining this research.

Revision: The method section has added content on propensity score matching. Added propensity score matching analysis results in the Results section, supplemented a detailed description of research limitations and bias control measures in the Discussion section, and all modifications are marked in red in the manuscript.

Attachment Submitted filename: Response to Reviewers 0520.pdf

# Peer review: Decision Letter 4

**DOI:** 10.1371/journal.pone.0353186.r009

**Authors:** Robert Chen, Robert Chen, Robert Chen, Robert Chen, Robert Chen

21 Jun 2026

Fascia Iliaca Compartment Block for Surgical Analgesia in Children with Osteogenesis Imperfecta: A Retrospective Cohort Study

PONE-D-25-58723R4

Dear Dr. Jiang,

We’re pleased to inform you that your manuscript has been judged scientifically suitable for publication and will be formally accepted for publication once it meets all outstanding technical requirements.

Within one week, you’ll receive an e-mail detailing the required amendments. When these have been addressed, you’ll receive a formal acceptance letter and your manuscript will be scheduled for publication.

An invoice will be generated when your article is formally accepted. Please note, if your institution has a publishing partnership with PLOS and your article meets the relevant criteria, all or part of your publication costs will be covered. Please make sure your user information is up-to-date by logging into Editorial Manager at Editorial Manager® and clicking the ‘Update My Information' link at the top of the page. For questions related to billing, please contact billing support.

If your institution or institutions have a press office, please notify them about your upcoming paper to help maximize its impact. If they’ll be preparing press materials, please inform our press team as soon as possible -- no later than 48 hours after receiving the formal acceptance. Your manuscript will remain under strict press embargo until 2 pm Eastern Time on the date of publication. For more information, please contact onepress@plos.org.

Kind regards,

Robert Jeenchen Chen, MD, MPH, ChFC®, EA

Academic Editor

PLOS One

Additional Editor Comments (optional):

Reviewers' comments:

Reviewer's Responses to Questions

**Comments to the Author**

1. If the authors have adequately addressed your comments raised in a previous round of review and you feel that this manuscript is now acceptable for publication, you may indicate that here to bypass the “Comments to the Author” section, enter your conflict of interest statement in the “Confidential to Editor” section, and submit your "Accept" recommendation.

Reviewer #1: All comments have been addressed

Reviewer #2: All comments have been addressed

**********

2. Is the manuscript technically sound, and do the data support the conclusions?

The manuscript must describe a technically sound piece of scientific research with data that supports the conclusions. Experiments must have been conducted rigorously, with appropriate controls, replication, and sample sizes. The conclusions must be drawn appropriately based on the data presented.

Reviewer #1: Yes

Reviewer #2: Partly

**********

3. Has the statistical analysis been performed appropriately and rigorously?

Reviewer #1: Yes

Reviewer #2: I Don't Know

**********

4. Have the authors made all data underlying the findings in their manuscript fully available?

The PLOS Data policy requires authors to make all data underlying the findings described in their manuscript fully available without restriction, with rare exception (please refer to the Data Availability Statement in the manuscript PDF file). The data should be provided as part of the manuscript or its supporting information, or deposited to a public repository. For example, in addition to summary statistics, the data points behind means, medians and variance measures should be available. If there are restrictions on publicly sharing data—e.g. participant privacy or use of data from a third party—those must be specified.

Reviewer #1: Yes

Reviewer #2: No

**********

5. Is the manuscript presented in an intelligible fashion and written in standard English?

PLOS ONE does not copyedit accepted manuscripts, so the language in submitted articles must be clear, correct, and unambiguous. Any typographical or grammatical errors should be corrected at revision, so please note any specific errors here.

Reviewer #1: Yes

Reviewer #2: Yes

**********

6. Review Comments to the Author

Please use the space provided to explain your answers to the questions above. You may also include additional comments for the author, including concerns about dual publication, research ethics, or publication ethics. (Please upload your review as an attachment if it exceeds 20,000 characters)

Reviewer #1: The authors have adequately addressed my comments raised in a previous round of review. The topic is relevant and important. Data support the conclusions.

Reviewer #2: Thanks for understanding the viewpoint and addressing the inherent bias. The purpose was to make sure that readers should look at the results with limitations related to the study design. By making changes in different versions and now adding propensity score at least major concerns are addressed.

**********

7. PLOS authors have the option to publish the peer review history of their article (what does this mean?). If published, this will include your full peer review and any attached files.

If you choose “no”, your identity will remain anonymous but your review may still be made public.

**Do you want your identity to be public for this peer review?**  For information about this choice, including consent withdrawal, please see our Privacy Policy.

Reviewer #1: No

Reviewer #2: **Yes:** Jamshed Akhtar

**********

# Editor report: Acceptance letter

**DOI:** 10.1371/journal.pone.0353186.r010

**Authors:** Robert Chen, Robert Chen, Robert Chen, Robert Chen, Robert Chen

PONE-D-25-58723R4

PLOS One

Dear Dr. Jiang,

I'm pleased to inform you that your manuscript has been deemed suitable for publication in PLOS One. Congratulations! Your manuscript is now being handed over to our production team.

At this stage, our production department will prepare your paper for publication. This includes ensuring the following:

* All references, tables, and figures are properly cited

* All relevant supporting information is included in the manuscript submission,

* There are no issues that prevent the paper from being properly typeset

You will receive further instructions from the production team, including instructions on how to review your proof when it is ready. Please keep in mind that we are working through a large volume of accepted articles, so please give us a few days to review your paper and let you know the next and final steps.

Lastly, if your institution or institutions have a press office, please let them know about your upcoming paper now to help maximize its impact. If they'll be preparing press materials, please inform our press team within the next 48 hours. Your manuscript will remain under strict press embargo until 2 pm Eastern Time on the date of publication. For more information, please contact onepress@plos.org.

You will receive an invoice from PLOS for your publication fee after your manuscript has reached the completed accept phase. If you receive an email requesting payment before acceptance or for any other service, this may be a phishing scheme. Learn how to identify phishing emails and protect your accounts at https://explore.plos.org/phishing.

If we can help with anything else, please email us at customercare@plos.org.

Thank you for submitting your work to PLOS ONE and supporting open access.

Kind regards,

PLOS ONE Editorial Office Staff

on behalf of

Dr. Robert Jeenchen Chen

Academic Editor

PLOS One
